# AXL inhibition improves BRAF-targeted treatment in melanoma

**DOI:** 10.1038/s41598-022-09078-z

**Published:** 2022-03-24

**Authors:** Marta Nyakas, Karianne Giller Fleten, Mads Haugland Haugen, Nikolai Engedal, Christina Sveen, Inger Nina Farstad, Vivi Ann Flørenes, Lina Prasmickaite, Gunhild Mari Mælandsmo, Kotryna Seip

**Affiliations:** 1 Department of Tumor Biology, Institute for Cancer Research, Oslo University Hospital, The Norwegian Radium Hospital, Ullernchausseen 70, 0379 Oslo, Norway; 2 Department of Oncology, Oslo University Hospital, The Norwegian Radium Hospital, Oslo, Norway; 3 Institute of Clinical Medicine, University of Oslo, Oslo, Norway; 4 Department of Pathology, Division of Laboratory Medicine, Oslo University Hospital, Oslo, Norway; 5 Department of Pathology, Oslo University Hospital, The Norwegian Radium Hospital, Oslo, Norway; 6 Department of Medical Biology, Faculty of Health Sciences, UiT/The Arctic University of Norway, Tromsø, Norway

**Keywords:** Cancer, Cell biology, Molecular biology, Molecular medicine, Oncology

## Abstract

More than half of metastatic melanoma patients receiving standard therapy fail to achieve a long-term survival due to primary and/or acquired resistance. Tumor cell ability to switch from epithelial to a more aggressive mesenchymal phenotype, attributed with AXL^high^ molecular profile in melanoma, has been recently linked to such event, limiting treatment efficacy. In the current study, we investigated the therapeutic potential of the AXL inhibitor (AXLi) BGB324 alone or in combination with the clinically relevant BRAF inhibitor (BRAFi) vemurafenib. Firstly, AXL was shown to be expressed in majority of melanoma lymph node metastases. When treated ex vivo, the largest reduction in cell viability was observed when the two drugs were combined. In addition, a therapeutic benefit of adding AXLi to the BRAF-targeted therapy was observed in pre-clinical AXL^high^ melanoma models in vitro and in vivo. When searching for mechanistic insights, AXLi was found to potentiate BRAFi-induced apoptosis, stimulate ferroptosis and inhibit autophagy. Altogether, our findings propose AXLi as a promising treatment in combination with standard therapy to improve therapeutic outcome in metastatic melanoma.

## Introduction

The incidence of malignant melanoma increases worldwide^1^. Whereas most patients with primary melanomas are cured by surgery alone^2^, patients with distant metastases require systemic treatment and historically their prognosis has been dismal with median survival of less than one year^3^. Although metastatic melanoma is still a potentially fatal disease, novel therapeutic options, including inhibitors targeting mutated BRAF and downstream MEK signaling, and the immune checkpoint inhibitors CTLA4 and PD-1 have resulted in improved overall survival (OS)^4–6^. Vemurafenib targets the most abundant BRAF^V600E^ gene mutation with good but short lived (six to nine months) initial response^7^. When combined with MEK inhibitors, a rapid onset of response and tumor debulking is often observed, but many patients become nonresponsive within a year^8^. Patients receiving immunotherapies with PD-1 inhibitor alone or in combination with CTLA4 inhibitor have five years OS of 33–52%^4,9^. Despite the high efficacy, approximately 40–65% and > 70% of patients demonstrate resistance towards anti-PD-1 and anti-CTLA4 therapies, respectively^4,10,11^.

Resistant tumors may arise under selective pressure of therapy from pre-existing subclones (intrinsic/innate resistance) or from an evolutionary process during treatment (acquired/adaptive resistance). There are numerous mechanisms underlying resistance towards BRAF inhibition, including genetic alterations, altered activity of signaling molecules or metabolic rewiring (reviewed in^12^). Recently, epithelial-to-mesenchymal transition (EMT) has been associated with drug resistance in various cancers^13^, including melanoma^14^. In melanoma tumor cells show intrinsic resistance to BRAF inhibition by sustaining a de-differentiated, mesenchymal-like, MITF^low^/AXL^high^ transcriptional profile^15^, or may switch towards this phenotype upon MAPK pathway inhibition^16^.

AXL is a member of the TAM (TYRO3, AXL and MERTK) tyrosine kinase receptor family, that regulates different aspects of cell proliferation, EMT, migration and regulation of immune responses^17^. AXL activation leads to downstream signaling through the PI3K/AKT, MAPK/ERK and STAT3 pathways^18–20^. AXL is highly expressed in many cancer types e.g. lung, breast and pancreas^21–23^ and is linked with tumor aggressiveness and resistance in different cancer types^24,25^, including melanoma^26^. The critical role of AXL makes it a promising target for cancer therapy.

BGB324 (R428, bemcentinib) is a highly potent new anti-cancer compound that blocks AXL autophosphorylation, leading to inhibition of cancer cell proliferation, invasion and metastasis^27,28^. BGB324 can also overcome resistance^23,29–31^, and enhance the effect of both chemotherapy and targeted drugs^32,33^. The exact molecular responses to AXL inhibitors are still elusive, although, it has been demonstrated that BGB324 can induce apoptosis^32,34^, affect lysosomal functions^34^ or abrogate autophagic flux^35^. Autophagy, a process where a cell degrades its own components via lysosomes, has a dual role in tumor development. It has been linked with both tumor suppression and promotion and may either counteract or promote drug-induced death depending on the context (reviewed in^36^). Recently, AXL signaling was also found involved in ferroptosis, a form of programmed cell death primed by iron and lipid hydroperoxides^37^.

In the current study we investigated the effect of BGB324 alone or in combination with vemurafenib as a potential new treatment strategy in melanoma. We have shown a therapeutic benefit of adding AXLi to the BRAF-targeted therapy in pre-clinical models in vitro and in vivo and identified apoptosis, autophagy and ferroptosis as cellular mediators of AXLi effects.

## Results

### Majority of melanoma lymph node metastases express AXL

**Figure 1:**
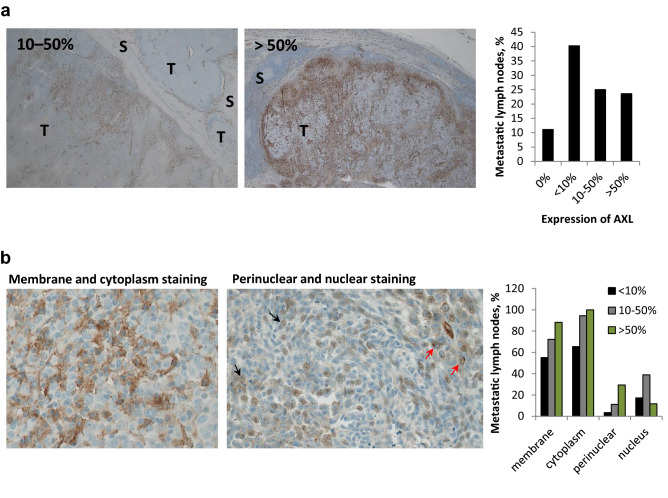
AXL expression and localization in melanoma lymph node metastases. AXL expression levels in 72 samples of metastatic lymph nodes were evaluated and depending on the expression status assigned into four sub-groups: no expression (0%), lower than 10%, 10–50% and more than 50%. (**a**) Representative IHC stainings of AXL expression level and localization are presented. Bar graph indicates the % of samples within each sub-group, where a total sample number was set to 100%. (**b**) Representative IHC staining of AXL within tumor cells, showing membrane and cytoplasm staining (left), and perinuclear and nuclear staining, indicated with red and black arrows, respectively (right). Bar graph indicates the % of AXL positive samples in the indicated cellular compartments within the different scoring sub-groups presented in (**a**). The total number within each sub-group exceeds 100% since samples may be positive in multiple cellular compartments. T, tumor region; S, stromal region. Images were taken at ×100 (**a**) or 200x (**b**) magnification.

To examine the relevance of AXL as a target in melanoma, its expression level was evaluated by immunohistochemistry (IHC) in 72 metastatic lymph node lesions. Most of the samples expressed AXL, where only eight metastases (11%) did not show any positive staining (Fig. 1a). 40% of all samples had lower than 10%, while approximately 25% had either 10–50% or more than 50% AXL expression. Regardless of the overall AXL expression levels, the protein was found to be heterogeneously expressed, showing positive staining in both the tumor periphery and its central areas. Importantly, AXL was primarily expressed by the metastatic cells and hardly by lymphatic tissue. When analyzing tumor cells, AXL staining was detected predominantly in the cell membrane and cytoplasm. In a few cases it was also detected in nucleus and as a perinuclear dot representing the Golgi area (Fig. 1b). When using 10% positive tumor cells as dichotomization criteria, regardless of intracellular localization, no association between AXL expression in the lymph node metastasis and any clinicopathological parameters registered from the primary tumor was detected (Supplementary Table S1). Neither was the AXL expression associated with overall or relapse-free survival. Overall, AXL was shown to be primarily expressed in metastatic melanoma cells, making AXL an interesting target for therapy.

### AXL inhibition shows a therapeutic effect in melanoma tissue ex vivo

**Figure 2:**
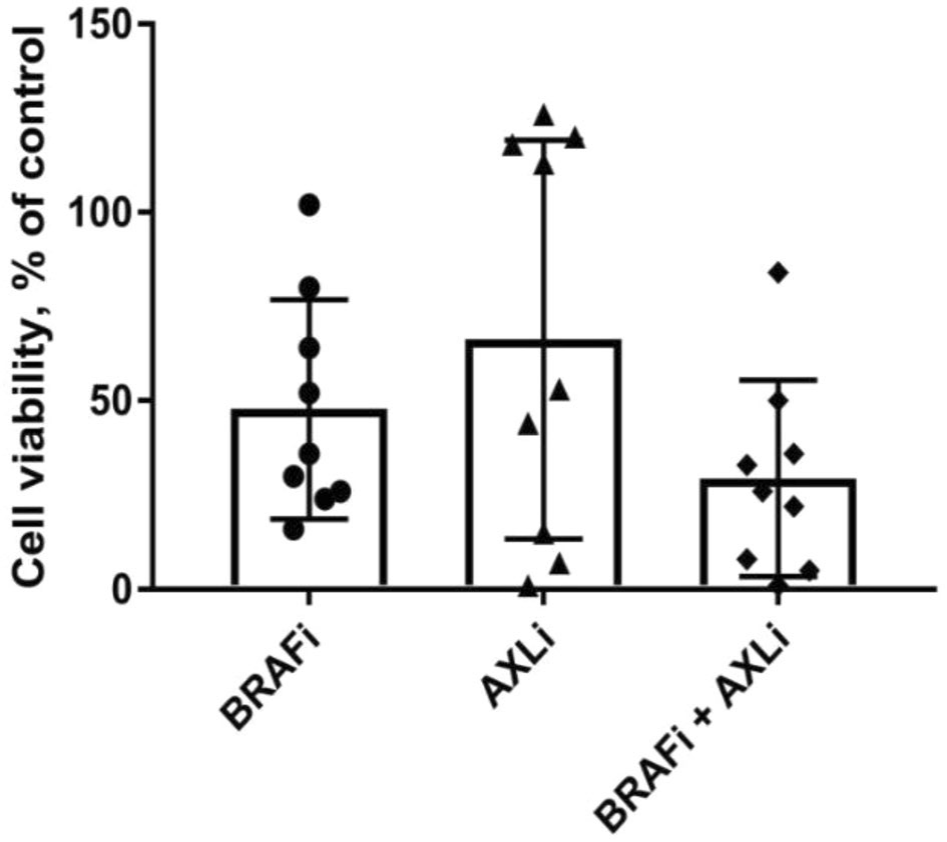
Ex vivo response to AXLi treatment in disaggregated cells from melanoma lymph node metastases. Cell viability of lymph node metastases cultured ex vivo and treated with 2 µM BRAFi, 3 µM AXLi or a combination of both drugs for five days. The treatment effect was scored by CTG assay and presented as % relative to the non-treated control set to 100% (Average ± SEM, n = 9).

To evaluate AXL inhibition as an anti-melanoma strategy, treatment response was examined on melanoma tissue from nine patients, who have not received prior treatment, using an ex vivo drug sensitivity assay^38^. Biopsies from BRAF mutated lymph node metastases were collected, disaggregated and treated with AXL inhibitor (AXLi) BGB324, BRAF inhibitor (BRAFi) vemurafenib or a combination of both. Most of the samples responded to BRAFi whereas varying response to AXLi was observed: four patient samples did not respond, two samples showed moderate response and in three samples treatment effect was more than 80% (Fig. 2). The response heterogeneity could be related to differences in AXL expression that was not addressed in this experiment. When combination treatment was applied, the mean viability in the patient tumor samples was reduced compared to either of the mono-treatments. Although very few patient samples were tested and statistical significance not reached, the results suggested that AXLi, alone or in combination with BRAFi, is a beneficial approach worth further investigation.

### Melanoma cells show reduced cell viability following combined inhibition of AXL and BRAF

**Figure 3:**
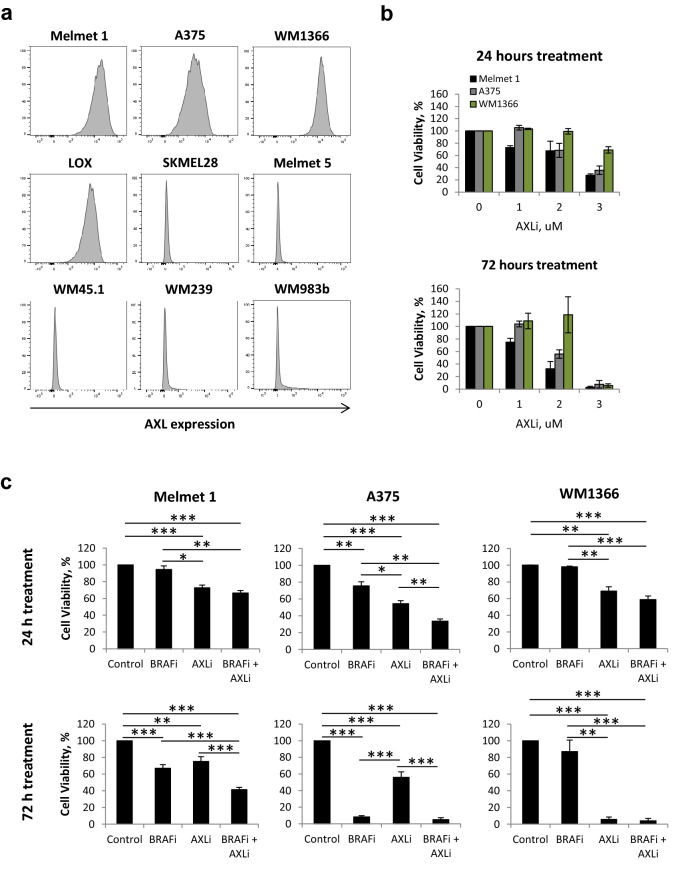
Melanoma cells show reduced cell viability following combined inhibition of AXL and BRAF. (**a**) The level of AXL (detected by flow cytometry) in nine melanoma cell lines. (**b**, **c**) Cell viability of three AXL^high^ melanoma cell lines treated with different concentrations, as indicated, of AXLi (**b**) or 2 µM BRAFi, 1 µM AXLi (for Melmet 1), 2 µM AXLi (for A375), 3 µM AXLi (for WM1366) or a combination of both drugs (**c**) for 24 or 72 h. The treatment effect was scored by CTG assay and presented as % relative to the respective non-treated controls set to 100%. Average ± SEM, n = 3. *p ≤ 0.05; **p ≤ 0.01; and ***p ≤ 0.001.

To further investigate AXL targeting and its induced biological mechanisms, a panel of nine melanoma cell lines was employed and screened for expression of AXL. Four cell lines, Melmet 1, A375, WM1366 and LOX were shown to express high levels, while the other five: SKMEL28, Melmet 5, WM45.1, WM239 and WM983B were shown to have low levels (Fig. 3a). To investigate the effect of AXLi alone or in combination with BRAFi, three cell lines with high expression of AXL, Melmet 1, A375 (both BRAF-mutated) and WM1366 (BRAF wild type), were selected for further studies. Following treatment with AXLi, all cell lines responded by reducing their metabolic activity that reflects reduced cell viability/proliferation. Melmet 1 was the most sensitive, while WM1366 was the least (Fig. 3b). To note, we have previously showed that AXL negative melanoma cells do not respond to the AXLi at similar doses as used here^39^.

When all three cell lines were treated with BRAFi, both Melmet 1 and A375 responded, and the latter was the most sensitive. WM1366 cells were, as expected, not responsive to BRAFi (Fig. 3c). Importantly, the most significantly pronounced and synergistic effects on cell viability were observed when the two drugs were combined and applied for either 24 (for A375) or 72 (for Melmet 1) hours. No additional effect of combined treatment was detected in WM1366 cells (Fig. 3c, Supplementary Fig. S1).

**Figure 4:**
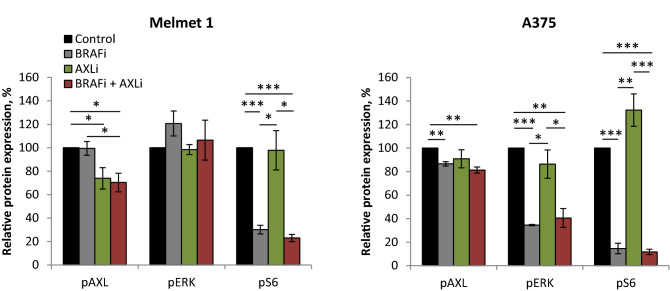
Melanoma cells show response to BRAFi and AXLi. Expression levels of proteins measured by FLOW (recording ≥ 2.5 × 10^4^ cells) in the differently treated Melmet 1 and A375 cells (2 µM BRAFi, 2 µM AXLi or a combination of both, DMSO for control) for 48 h and presented as relative to respective non-treated controls set to 100%. Average of signal median ± SEM, n = 3. *p ≤ 0.05; **p ≤ 0.01; and ***p ≤ 0.001.

At the molecular level (Fig. 4, Supplementary Fig. S2) we observed a decrease in phosphorylation of AXL (pAXL) in Melmet 1 and A375 48 h after AXLi treatment, although the effect was statistically significant only for the Melmet 1 cells. Despite a non-significant decrease in pAXL in A375 (that could be related to the absence of AXL-stimulation with e.g. GAS6 ligand) we concluded that both cell lines suppress AXL signaling in the presence of AXLi. Response to BRAFi was observed as a pronounced reduction in phosphorylated ERK (pERK) in the A375 cells, while pERK was not affected in the Melmet 1 cell line. The lack of effect on pERK was a robust phenomenon in Melmet 1 and could be explained by previously reported recovery of pERK yet keeping the growth arrest in BRAF^V600^-mutated cells^40^. This was not investigated further, since we observed a strong reduction in phosphorylation of one of the downstream mTORC1 substrates, protein S6, another good indicator of general response to BRAFi^41^. Reduced phosphorylation of S6 (pS6) was observed in both cell lines after treatment with BRAFi mono-treatment or in combination with AXLi. Expression of total AXL was decreased upon BRAFi treatment, while total ERK was shown to be little affected by different treatments (Supplementary Fig. S2). Altogether, this suggests that the three tested melanoma cell lines show good response to AXLi, and that their sensitivity to BRAFi depends on the BRAF mutation status. The best response, in line with the ex vivo assay, was observed when the two drugs were combined in BRAF mutated cells.

### AXLi enhances apoptosis when combined with BRAFi in vitro

**Figure 5:**
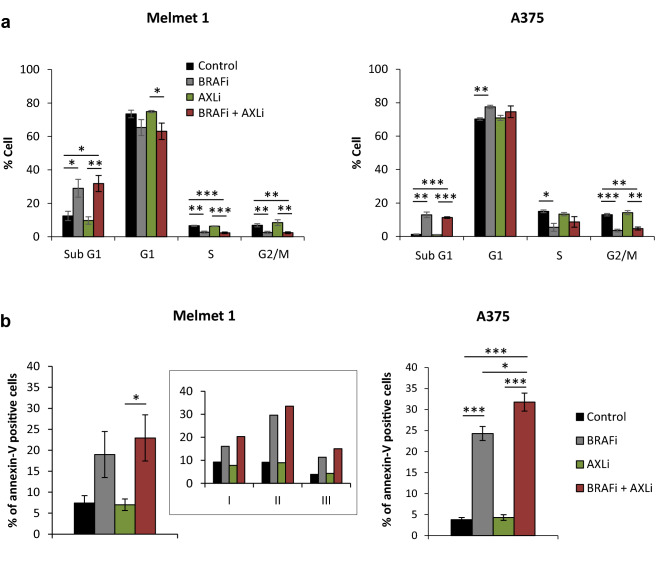
Apoptosis induction in melanoma cells following combined inhibition of AXL and BRAF. Melmet 1 and A375 cells were treated with 2 µM BRAFi, 2 µM AXLi or a combination of both, DMSO for control for 48 h, stained with Hoechst (**a**) or annexin-V (**b**) and analyzed by flow cytometry. Insert in (**b**) represents the % of annexin-V positive Melmet 1 cells from three independent biological experiments. Average ± SEM, n = 3. *p ≤ 0.05; **p ≤ 0.01; ***p ≤ 0.001.

To understand whether the observed loss of viability was due to reduced proliferation and/or increased apoptosis, we first analyzed the treatment-induced effect on cell cycle distribution in Melmet 1 and A375 after 48 h. As shown in Fig. 5a, AXLi alone had no significant effects on cell cycle distribution. BRAFi mono-treatment, however, reduced cell numbers in S and G2/M phases and slightly affected the G1 phase, suggesting impaired cell proliferation. We also observed a significant increase in cell number in the sub-G1 phase (representing DNA fragmentation), an indicator of apoptosis, after the treatment with BRAFi. Even though the cell lines showed higher sensitivity for the combined treatment compared to any of the mono-treatments (Fig. 3c), no clear additional effects on cell cycle distribution were observed.

Since DNA fragmentation is a late event in apoptosis^42^, a more sensitive and specific approach, annexin-V staining^43^, was employed to evaluate effects on apoptosis (Fig. 5b). A significant increase in annexin-V positive cells was observed in both cell lines after BRAFi mono-treatment, suggesting induced apoptosis. Such effect was not seen with AXLi. When the two drugs were combined, an additional increase in annexin-V positive cell fraction was detected. This reached statistical significance only in the A375 cells, although, an increase in annexin-V positive cells also was observed in all three individual Melmet 1 experiments. Altogether, our data suggest that potentiated apoptosis contributes to reduced cell survival following combined inhibition of AXL and BRAF.

### Combined inhibition of AXL and BRAF increases the anti-tumor effect in vivo and is associated with reduced expression of anti-apoptotic proteins

**Figure 6:**
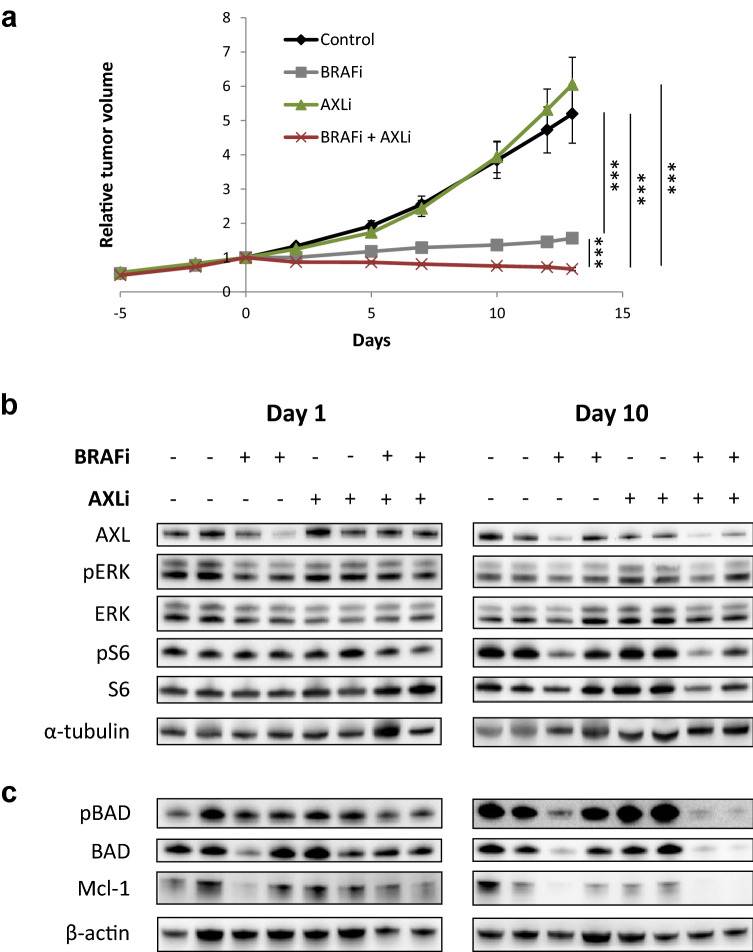
Melanoma cells in vivo show anti-tumor effect and apoptosis following combined inhibition of AXL and BRAF. Subcutaneous Melmet 1 tumors were treated with BRAFi (12.5 mg/kg), AXLi (50 mg/kg), the combination or vehicle (DMSO/methylcellulose) twice/day for 14 days. The tumor volume over time, presented as relative to the volume at treatment initiation (**a**) and the levels of indicated protein related to treatment efficacy (**b**) and apoptosis (**c**) in two tumors from each treatment group at day 1 and 10 are presented (cropped gels). (**a**) Average ± SEM, n = 8, ***p ≤ 0.001.

To evaluate the effect of the combination treatment in vivo, Melmet 1 xenografts were established and treated with AXLi, BRAFi or a combination of both, twice daily for 14 days (Fig. 6a). AXLi alone did not inhibit tumor growth at the dose used, while growth was significantly inhibited by BRAFi mono-treatment. Compared to either one of the mono-treatments, a significant regression in tumor size was observed when xenografts were treated with the AXLi and BRAFi combination. Following treatment withdrawal, all tumors regrew. However, tumors subjected to the combined treatment had slightly slower regrowth rate, possibly due to a lower number of cells left after the treatment (Supplementary Fig. S3).

At the molecular level a variation in protein expression between parallel samples was observed, but as expected, a decrease in pERK and pS6 in BRAFi treated tissue harvested at days 1 (pERK only) and 10 were detected (Fig. 6b, Supplementary Fig. S4). In the current experiment we were not able to detect any pAXL signal, but in line with previous observations^39^, a slight increase in total AXL was observed at day 1 when treated with AXLi alone. This is, however, counteracted at day 10 where reduced expression of total AXL is observed. To test whether apoptosis was induced in the differently treated tumors, tissue samples were analyzed for cleaved-caspase 3. However, neither western blot nor IHC detected this protein. Other proteins related to the intrinsic apoptosis pathway, including total and phosphorylated BAD and Mcl-1, were down-regulated after combination treatment, suggesting activation of apoptosis, while remained unaffected by any of the mono-treatments (Fig. 6c, Supplementary Fig. S4). In summary, the combined treatment significantly inhibited tumor growth, and this was associated with reduced expression of anti-apoptotic proteins.

### Proteome alterations in AXLi- and BRAFi-induced tumor growth inhibition

**Figure 7:**
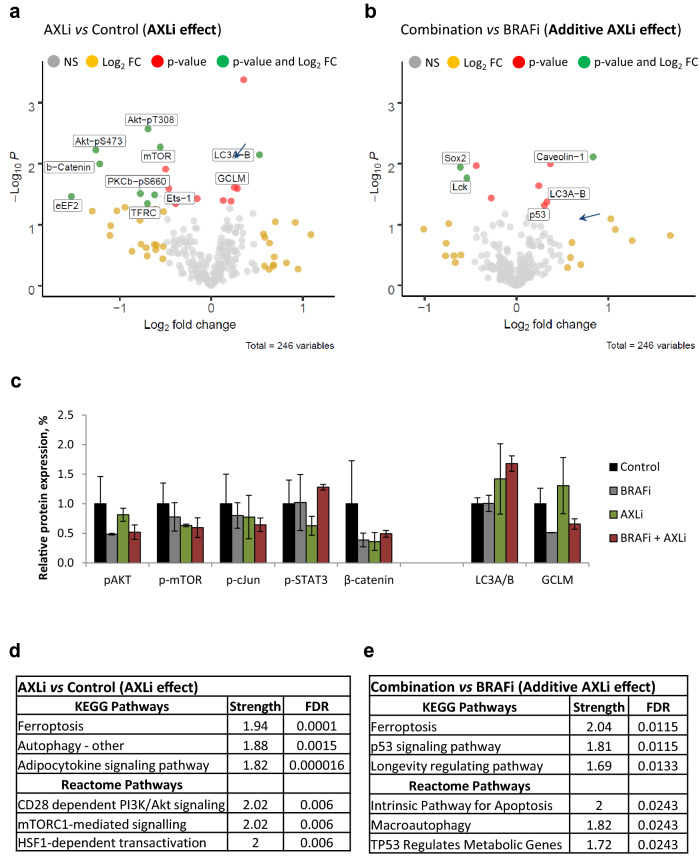
Effects of AXLi mono- and additive treatments. Ten days (twice/day) treated Melmet 1 tumors (BRAFi: 12.5 mg/kg, AXLi: 50 mg/kg, the combination or vehicle (DMSO/methylcellulose)) were subjected to RPPA analysis and AXLi-induced effects on protein expression either upon mono-treatment “AXLi *vs.* control” (**a**) or as an additive effect when combined with BRAFi “Combination *vs* BRAFi” (**b**) presented as volcano plots. Differentially expressed proteins: only *p* value < 0.05 (red), only log2 fold change difference (log_2_FC) > 0.5 (yellow) and both *p* value < 0.05 and log_2_FC > 0.5 (green). Not significantly (NS) changed proteins indicated in grey. Positive and negative log_2_FC values represent increased and decreased protein expression, respectively, in AXLi *vs* Control or AXLi + BRAFi *vs* BRAFi treated tumors. (**c**) Expression levels of proteins (after normalization to the loading control, α-tubulin) measured by SWI in the treated tumors as indicated. Average ± St.Dev., n = 2. (**d**, **e**) Top three identified functional enrichments (ranked based on strength set to be above 1.5) within KEGG^65^ and Reactome signaling pathway databases, analyzed by STRING (www.string-db.org), in AXLi- mono-treated (**d**) or as a part of combination with BRAFi (**e**). Strength and False Discovery Rate (FDR) describe how large and significant, respectively, the enrichment effect is.

To reveal other potential AXLi-induced mechanisms, protein lysates from in vivo treated Melmet 1 tumors were subjected to RPPA analysis to assess expression of 246 proteins, of which 65 were in their phosphorylated state. A higher number of significantly differently expressed proteins were detected at day 10 than day 1, and day 10 was chosen as a time point for further analysis. To investigate AXLi-induced effects on tumor cells either upon mono-treatment or in combination with BRAFi, further referred to as additive AXLi effect, two treatment groups were analyzed: “AXLi *vs* control” and “Combination *vs* BRAFi”, respectively. A higher number of significantly differently expressed proteins were identified upon AXLi mono-treatment than as an AXLi additive effect (Fig. 7a, b and Supplementary Fig. S5). Among several AXLi down-regulated proteins were proteins involved in AKT, mTOR, JNK, STAT3, and β-catenin signaling pathways. The latter result is in line with previous studies^31,44,45^, and was also validated by Simple Western Immunoassay (SWI) (Fig. 7c, Supplementary Fig. S6). Even though most of the validated AXLi down-regulated proteins were also down-regulated by BRAFi alone (Fig. 7c), the ferroptosis and autophagy associated markers, glutamate-cysteine ligase (GCLM) and Light Chain 3 (LC3) (A and B isoforms), were shown to be upregulated by AXLi mono- or combination treatment (LC3A/B only) (Fig. 7a–c).

To identify functional associations between treatment-modulated protein expression, the RPPA data were subjected to the pathway enrichment analysis. Among the top three AXLi-affected pathways in mono-treated cells were autophagy and PI3K/AKT/mTOR signaling, with mTORC1 being the master negative regulator of autophagy^46^ (Fig. 7d). Not surprisingly, intrinsic pathways of apoptosis and autophagy were also among the top three signaling pathways associated with the additive AXLi effect (Fig. 7e). To be noted, autophagy was among the enriched functions also in BRAFi treated cells (Supplementary Table S2), even though we did not detect LC3A/B up-regulation (Fig. 7c). Ferroptosis, however, was identified as the most enriched pathway upon AXLi mono- and additive effects (Fig. 7d, e), but was not associated with the BRAFi mono-therapy, nor with the additive BRAFi effect when the two drugs were applied (Supplementary Table S2). Altogether, this suggests that AXLi affects proteins involved in both autophagy and ferroptosis, but that only ferroptosis represents an AXLi-specific cellular response.

### AXL inhibition reduces autophagic flux at a late stage in the autophagy pathway

**Figure 8:**
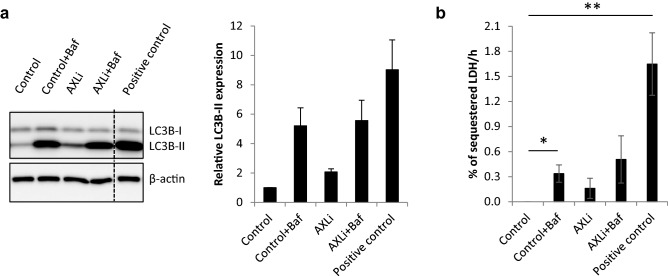
AXLi limits autophagic flux at a late stage in the autophagy pathway. Melmet 1 cells were treated with 1 µM AXLi for 48 h with or without co-treatment with 100 nM lysosomal inhibitor Bafilomycin A1 (Baf) for the 3 final hours of the treatment (DMSO for control). As a positive control, cells were in addition to Baf subjected to amino acid starvation by incubation in EBSS medium for 3 h. (**a**) Expression levels of indicated proteins in the differently treated cells. A representative western image is shown (cropped gel). Quantified expression levels of LC3B-II (after normalization to the loading control, β-actin) presented as relative to non-treated control set to 1. Average ± St. Dev, n = 2. (**b**) The LDH sequestered assay was performed and the results presented as the mean LDH sequestration activities (in %/h) in differently treated cells, normalized to the control set to 0. Average ± SEM, n = 3, *p ≤ 0.05; **p ≤ 0.01.

To assess the effect of AXLi on autophagic activity in more detail, we performed two classical autophagy assays: (1) LC3B immunoprobing in cells that had been treated in the absence or presence of lysosomal inhibitor Bafilomycin A1 (Baf) to determine autophagic membrane flux^47^, and (2) measurement of lactate dehydrogenase (LDH) activity in sedimentable cellular fractions with/without Baf to determine autophagic cargo flux^47,48^ (see Supplementary Fig. S7 for an explanation of the assay principles). Upon lipid conjugation, the cytosolic form of LC3B (LC3B-I) is converted to the membrane-bound form (LC3B-II), which stays attached to the autophagic membranes throughout the pathway until autophagosome-lysosome fusion takes place and whereupon LC3B-II is degraded (Supplementary Fig. S7). The latter can be blocked by Baf, upon which elevated levels of LC3B-II can, therefore, reflect the degree of LC3B flux through the autophagy pathway. LC3B flux was readily detectable in Melmet 1 cells, as Baf induced a strong increase in LC3B-II levels (Fig. 8a, Supplementary Fig. S8). Interestingly, increased levels of LC3B-II were also observed when cells were treated with AXLi alone (Fig. 8a), whereas the Baf-induced increase in LC3B-II was similar when comparing AXLi + Baf-treated cells *vs* Control + Baf-treated cells (Fig. 8a). This indicates that AXLi has no effect on autophagic flux activity up until the late stage of autophagy, where AXLi reduces the flux.

Autophagy is not always accompanied by LC3B flux^47,49,50^. Therefore, we next assessed autophagic cargo flux by the LDH sequestration assay, which measures the uptake of the cytosolic enzyme LDH into autophagosomes (Supplementary Fig. S7). As expected, increased LDH sequestration was detected upon Baf treatment (Fig. 8b). A tendency of increased LDH sequestration was also observed in cells treated with AXLi alone (Fig. 8b), suggesting a partial block in autophagic cargo flux at a late stage of autophagy. Importantly, no significant difference in Baf-induced LDH sequestration was detected when AXLi + Baf-treated cells were compared to Control + Baf sample (Fig. 8b), indicating that AXLi treatment does not increase autophagic sequestration activity. In summary, the results obtained with the LC3B flux and LDH sequestration assays are in line with each other and indicate that AXLi-treatment imposes a partial reduction in autophagic flux at a late stage in the autophagy pathway.

### AXL inhibition induces ferroptosis

**Figure 9:**
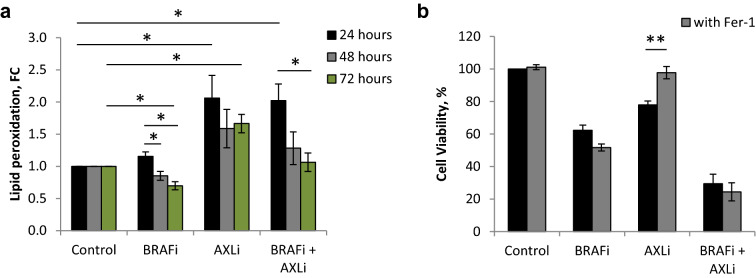
Ferroptosis induction in melanoma cells following inhibition of AXL. (**a**) Lipid peroxidation measured by FLOW (recording ≥ 1 × 10^4^ cells) in differently treated Melmet 1 cells (2 µM BRAFi, 1 µM AXLi or a combination of both, DMSO for control) for 24–72 h and presented as a fold change (FC) between the detected red signal of lipid peroxidation sensor in treated samples compared to the respective non-treated controls set to 1. (**b**) Cell viability of Melmet 1 cells treated with 0.25 µM ferroptosis inhibitor ferrostatin-1 (Fer-1), 2 µM BRAFi, 0.5 µM AXLi or a combination of two/three compounds, DMSO for control, for 72 h. The treatment effect was scored by CTG assay and presented as % relative to the respective non-treated controls set to 100%. Average ± SEM, n = 3. *p ≤ 0.05; **p ≤ 0.01.

Lipid peroxidation (LPO) is regarded as one hallmark of ferroptosis^51^. To determine whether AXLi affects LPO, we used a sensitive radiometric LPO sensor that changes its fluorescence from red to green upon peroxidation by ROS in cells. Due to Melmet 1 cells being GFP-tagged, only differences in intensity of the red color was tracked. This showed to be a sufficient measurement as a drop in red signal intensity was detected in both hydrogen peroxide (positive assay control) and RSL3- (ferroptosis inducer) treated cells (Supplementary Fig. S9). When cells were treated with AXLi alone a significant drop in red color intensity was also observed (Supplementary Fig. S9), indicating increased LPO (Fig. 9a). Interestingly, upon combination treatment with BRAFi, LPO induction was diminished with time, possibly due to the antagonistic effect of BRAFi on LPO (Fig. 9a). To examine further the role of ferroptosis in AXLi-reduced cell survival/proliferation, the ferroptosis inhibitor, ferrostatin-1, was tested on Melmet 1 cells treated with AXLi, BRAFi or a combination of both. Treatment with ferrostatin-1 alone had no effect on Melmet 1 cell viability throughout 72 h (Fig. 9b, Supplementary Fig. S10) and did not influence BRAFi mediated reduction in cell viability (Fig. 9b). When ferrostatin-1 was combined with AXLi, it completely restored cell viability (Fig. 9b). However, no rescue was observed when ferrostatin-1 was added to cells treated with the combination of AXLi and BRAFi (Fig. 9b), regardless of treatment duration or ferrostatin-1 doses (Supplementary Fig. S10). Altogether, these results implicate ferroptosis as a cellular response mechanism to AXL inhibition, however, its influence on response to the AXLi and BRAFi combination needs further clarification.

## Discussion

High levels of AXL is associated with the de-differentiated, mesenchymal cell state, which shows enhanced resistance to targeted therapy, like MAPK pathway inhibitors in malignant melanoma^26^ or EGFR inhibitors in lung cancer^21^. Consequently, AXL has been suggested as an attractive therapeutic target to eliminate such cells or sensitize them to other therapies. Here we employed pre-clinical melanoma models with high levels of AXL and investigated the anti-cancer effects and molecular mechanisms induced by the AXL inhibitor BGB324, when used alone and in combination with the BRAF inhibitor vemurafenib. The aim was to evaluate the therapeutic potential of such treatment strategies for improving melanoma treatment.

When the BRAF^V600E^ mutated, AXL^high^ melanoma cell lines, Melmet 1 and A375, were treated with AXLi and BRAFi combination, reduced cell survival was observed compared to the effect of mono-treatments. The calculated Bliss independence score revealed a synergistic interaction between the two drugs in both cell lines. Importantly, we also observed enhanced anti-tumor effects in vivo, where significant regression in tumor size was achieved in Melmet 1 xenografts treated with the drug combination compared to BRAFi alone. Taken together, this suggests that AXLi can potentiate BRAF-targeted therapy in various melanoma models. This is in line with lung cancer data where AXL inhibitors could enhance the effect of EGFR-targeted therapies in resistant AXL^high^ cells^21,52^.

One of the potential mechanisms behind enhanced anti-tumor effect when AXLi is combined with BRAFi, is induction of apoptosis. Both enrichment of the apoptotic cell fractions in vitro and down-regulation of anti-apoptotic proteins in vivo, point at increased apoptosis in combination treatment compared to BRAFi alone. This is in accordance with many studies, where AXLi enhanced apoptosis when combined with targeted-^32,53,54^, chemo-^55^ or immune-^39^ therapies. Altogether, this suggests a common molecular mechanism or several targets that leads to AXLi-induced potentiation of apoptosis regardless of the combination partner. We did, however, not detect increased apoptosis by AXLi alone, neither when analyzing in vitro treated cell lines nor tissue harvested from in vivo treated xenografts. This result contradicts the previous studies, which reported enhanced levels of apoptotic markers upon AXL inhibition^32^, or AXL downregulation^54^. We do not exclude the possibility that different treatment regimens or other apoptosis markers/assays might detect signs of apoptosis also in our experimental systems. However, our data strongly suggest that AXLi mono-treatment primarily induces another, non-apoptotic cell death mechanism, in our models. Specifically, we identified ferroptosis as a likely mediator of AXLi-induced cell growth inhibition.

We validated the involvement of ferroptosis by (1) increased LPO upon AXLi treatment and (2) rescuing AXLi-reduced Melmet 1 cell viability with a ferroptosis inhibitor. Inhibition of ferroptosis, however, did not rescue cells treated with BRAFi or combination of BRAFi and AXLi, possibly due to lower level of LPO compared to that observed with AXLi alone. This suggests that ferroptosis is not an additional death mechanism in cells that respond to BRAFi, which utilize BRAFi-induced death pathways, like apoptosis. The fact that AXLi plays a role in ferroptosis, however, can be highly beneficial for combination therapy where multiple cell death pathways could be activated. Recently, Tsoi et al.^56^ reported that AXL^high^, BRAFi-resistant melanomas are especially sensitive to ferroptosis, which appears to be a general phenomenon for AXL^high^ cancer cells^57^. Ferroptosis-inducers were shown to effectively target such cells^56,57^. On this basis, it was suggested that combination of ferroptosis-inducing drugs and BRAFi will target cells in opposite phenotypic states, preventing phenotypic plasticity, and enhancing the overall therapeutic effect^56^. It is tempting to speculate that AXLi, as a ferroptosis inducer, could act similarly when combined with BRAFi and applied to phenotypically heterogeneous tumors. Although ferroptosis is just recently discovered^58^, the influence of this death mechanism on the efficacy of different cancer treatments (chemo-, immune-, radio- and targeted therapies) has already been shown^59–62^. Consequently, ferroptosis-induction has been a suggested strategy to improve outcomes of different therapies, and several ferroptosis-directed clinical trials have been initiated during the last years (reviewed in^63^). The potential of combining AXLi with other therapies than BRAFi in melanoma, with a putative beneficial involvement of AXLi-stimulated ferroptosis is thus worthy of further study.

Pathway enrichment analysis of the proteome data pointed at autophagy as another biological process that is modulated by AXLi. This included indications of downregulated mTORC1 signaling and upregulated autophagic components like LC3A/B, suggesting a potential of AXLi to induce autophagy. However, measurements of autophagic membrane- (LC3B-II) and cargo (LDH) flux indicated that autophagy activity was not increased upon treatment with AXLi. On the contrary, both assays indicated that AXLi inhibits autophagic flux at a late stage in the autophagic pathway, leading to accumulation of LC3B-II and sequestered LDH. This is in accordance with results obtained in other cell types, where such AXLi effect was linked to the therapeutic outcome^34,35^. Although the observed autophagy-inhibitory effects were relatively moderate as compared to those obtained with the lysosomal inhibitor Baf, our data suggest that the influence of AXLi on autophagy may affect its therapeutic efficacy in melanoma, and this warrants further studies.

Overall AXL expression is quite common among malignant melanomas and the possibility to potentiate targeted therapy by AXL inhibition is thus highly attractive. We have shown that 89% of patients’ lymph node biopsies stained positive for AXL, and Sensi et al*.*^28^ reported that 40% of melanoma metastases of different origin expressed AXL. The high number of AXL positive lymph node metastases could be related to lymph nodes being the first metastatic location where disseminating, mesenchymal-phenotype cells settle. In concordance, Sensi et al. ^28^*.* detected a higher percentage of AXL positive cases among metastatic lesions (40%) than primary tumors (30%). Together this makes AXL a promising target in stage III and metastatic melanoma. The numerous clinical trials in various cancer types, including melanoma, testing AXLi as mono-treatment or in combination with chemo- (in pancreas cancer, NCT03649321), immuno- (in lung cancer, NCT03184571 and melanoma, NCT02872259) or targeted (in melanoma, NCT02872259) therapies reflects a promise in AXL as a therapeutic target.

In conclusion, our findings have shown that the AXL inhibitor BGB324 affects multiple cellular pathway and cell death mechanisms and represents an attractive approach for improving standard targeted treatment in malignant melanoma. By potentiating BRAFi-induced apoptosis, stimulating ferroptosis or inhibiting autophagy AXL inhibitors may offer new treatment possibilities for resistant tumors.

## Materials and methods

### Cell lines

Malignant melanoma cell lines Melmet 1 (derived from subcutaneous metastasis and stably labeled with green fluorescent protein (GFP)), Melmet 5 and LOX (both derived from lymph node metastases) were established from patients operated at the Norwegian Radium Hospital, Oslo University Hospital, Oslo, Norway. Informed consent was obtained from each patient before tissue acquisition and The Norwegian Regional Committees for Medical and Health Research Ethics (REK) approved the study (approval numbers: 2011/2183, S-01252, 2.2007.997). WM983b, WM239, WM45.1 and WM1366 were kindly provided from Dr. Meenhard Herlyn, The Wistar Institute, Philadelphia, PA. SKMEL28 and A375 were purchased from American Type Culture Collection, Manassas, VA. All melanoma cell lines were cultured in RPMI 1640 medium, supplemented with 5% fetal bovine serum and 2 mM L-Alanyl L-Glutamine (all from Sigma-Aldrich, St. Louis, MO), further referred as RMPI++ medium. For amino acid starvation, the cells were washed and incubated in Earle’s balanced salt solution (EBSS) (Thermo Fisher Scientific, 24010043). The cell cultures were maintained at 37 °C in a humidified atmosphere containing 5% CO_2_ and routinely tested for mycoplasma.

### Patient material

Paraffin-embedded tissue from 72 lymph node and subcutaneous metastatic melanomas and nine freshly harvested lymph node metastases were obtained from patients who underwent surgery at the Department for Plastic and Reconstructive Surgery, Oslo University Hospital, Oslo, Norway. Histologic diagnosis was based on World Health Organization criteria, and the pathologic staging was performed according to the tumor, node and metastatic classification system AJCC 7.0. Characteristics of patient material used for immunohistochemistry (IHC) are provided in Supplementary Table S1. Informed consent was obtained from each patient before tissue acquisition in accordance with the Declaration of Helsinki and approved by the Regional Committees for Medical and Health Research Ethics (approval numbers: 2014/2208 and 2015/2434). BRAF^V600^ and NRAS mutations were determined by routine diagnostics by in-house PCR based assay.

### Drugs

BRAF inhibitor vemurafenib (PLX4032) was purchased from Selleck Chemicals (Houston, TX), BGB324 (R428, bemcentinib) was kindly provided by BergenBio (Bergen, Norway). Ferroptosis inhibitor ferrostatin-1 and inducer RSL3 were purchased from Merck Life Science (Kenilworth, NJ). Bafilomycin A1 was from Enzo (Novi, MI). All drugs were dissolved in DMSO. For animal studies, the drugs were further diluted in 0.5% methylcellulose (Sigma-Aldrich).

### Cell viability assay

3000–4500 cells/well were grown for 24 h in white 96-well plates (Costar, Costar, NY) before 24–72 h treatments took place. Cell survival was evaluated by using CellTiter-Glo® (CTG) assay (Promega, Madison, WI) adding CTG directly to the wells (1:1). After 10 min, bioluminescence was measured by Victor™ X3 multiplate reader (Perkin Elmer, MA).

### Flow cytometry

Cells were fixed in 1.6% paraformaldehyde at room temperature (RT) for 15 min and permeabilized with 100% ice-cold methanol. For AXL expression analysis melanoma cell lines were stained with antibody against AXL (1H12) (kindly provided by BergenBio) for 30 min, followed by a washing step, before staining for 30 min with a secondary donkey anti mouse DyHigh 647 antibody (Jackson ImmunoResearch, West Grove, PA, #715-495-150). For phospho protein analysis differently treated samples were given a fluorescent “barcode” by adding pacific orange dye (Life Technologies, Carlsbad, CA) at different concentrations ranging from 0 to 2 ng/μL. After incubation at RT for 30 min, followed by washing, the barcoded samples were combined for simultaneous staining with antibodies against pAXL (MAB6965, Clone 713610, R&D, 2.5 µg/10^6^ cells) additionally conjugated for 30 min with a secondary Alexa 647 antibody (Invitrogen), pERK-PE (#14095, Clone 197G2, 1:50) and pS6-Alexa Fluor 647 (#4851, Clone D57.2.2E, 1:200) (both from cell signaling technologies) for 30 min. For cell cycle and apoptosis analysis: cells were stained with 20 µg/mL Hoechst 33342 (Invitrogen, Eugene, OR, #H3570) or Annexin V-Alexa Fluor 647 antibody (Invitrogen, Carlsbad, CA, #A23204) and propidium iodide (Sigma-Aldrich, #P4864) for 1 h at 37 °C or for 30 min at RT, respectively. The stained samples were analyzed on a LSR II flow cytometer controlled by BD FACS Diva™ software (BD Bioscience, San Jose, CA). The data was analyzed by using FlowJo software (FlowJo, Ashland, OR).

### Protein analysis

Protein lysates were prepared by re-suspending the cells from either in vitro studies or mechanically disintegrated frozen tumors in reverse-phase protein array (RPPA) lysis buffer (1% Triton X-100, 50 mM HEPES, 150 mM NaCl, 1.5 mM MgCl_2_, 1 mM EGTA, 100 mM NaF, 10 mM Na_4_P_2_O_7_, 1 mM Na_3_VO_4_, 10% glycerol) containing protease- and phosphatase inhibitors (Roche Applied Science, Mannheim, Germany) followed by ultrasonication. For Western blotting, total cellular proteins (10–20 µg) were separated on NuPAGE® Novex 4–12% Bis–Tris Gel (Invitrogen, Carlsbad, CA, #WG1402BOX) or 4–20% Criterion TGX Midi protein gels (for LC3B) (Bio-Rad, Hercules, CA, #5671094). The gels were blotted onto nitrocellulose or PVDF membranes using the iBlot™ system from Novex (Life Technologies, Kiryat Shmona, Israel, #IB301001) or a Criterion blotter with plate electrodes (Bio-Rad), respectively. The membranes were blocked either with 10% BSA or 5–7.5% dry milk TBST solution and incubated with primary antibodies at 4 °C overnight, followed by incubation with secondary antibodies at RT for 1 h. After application of Super Signal West Dura kit solution (Thermo Scientific, Rockford, IL), the membranes were visualized using GBox (Syngene, Cambridge, UK) and the GeneSnap software (Syngene, Cambridge, UK) or Chemi-Doc Imaging System (Bio-Rad, Hercules, CA).The following antibodies from Cell Signaling Technology (Danvers, MA) were used anti-AXL (#8661) 1:1000, anti-pERK (#4370) 1:2000, anti-ERK (#4695) 1:1000, anti-pS6 (#4858) 1:2000, anti-S6 (#2217) 1:1000, anti-pBAD (#4366) 1:1000, anti-BAD (#9292) 1:1000, anti-Mcl-1 (#4572) 1:1000, anti-LC3B (#2775) 1:1000. Anti-α-tubulin (#CP06) 1:5000 was from Merck Millipore (Burlington, MA) and anti-β-actin (A5316) 1:5000 was from Sigma-Aldrich.

The Simple Western Immunoassay (SWI) was performed on a PeggySue™ (ProteinSimple, San Jose, CA) applying 0.8 µg/µL protein lysate and using a 12–230 kDa size separation master kit according to the manufacturer`s instructions. All settings were kept on default, except primary antibody incubation time, which was set to 60 min. The Compass software (Protein Simple, version 2.7.1) was used to program the PeggySue instrument and for quantification of the results. The following antibodies from Cell Signaling Technology were used: anti-pAKT (#4060) 1:25, anti-p-mTOR (#5536) 1:50, anti-β-catenin (#19807) 1:50, anti-LC3A/B (#12741) 1:50, anti-p-cJun (#3270) 1:50, anti-pSTAT3 (#9145) 1:50, anti-ERK (#4695) 1:200, anti-AXL (#8661) 1:50. Anti-GCLM (ab126704) 1:50 was from abcam (Rozenburg, Netherlands) and an anti-β-actin (A5316) 1:200 was from Sigma-Aldrich.

The RPPA analysis on Melmet 1 tumors was performed at MD Anderson RPPA core facility (Houston, TX). Denatured tumor protein lysates were arrayed at serial dilutions on nitrocellulose-coated slides. First a validated primary antibody was used to probe each slide (specified at the core facility’s home page) then a biotin-conjugated secondary antibody followed; the signal was detected by a 3`3-diaminobenzidine (DAB) colorimetric reaction. MicroVigene software (VigeneTech, Carliste, MA) was used to scan the slides and to analyze spot intensities. Each dilution curve was fitted with a logistic model (“Supercurve Fitting”). After log2-transformation the relative protein values were normalized for protein loading and converted into linear values.

### Lactate dehydrogenase (LDH) sequestration assay

The LDH sequestration assay was performed as described previously^48^, with minor modifications. Briefly, Melmet 1 cells were allowed to settle for 24 h before 48 h treatment with AXLi (1 µM). Three hours before sample harvest, cells were subjected to 100 nM Bafilomycin A1 (Baf), a lysosomal inhibitor, or 0.1% DMSO vehicle. For a positive control, cells were additionally treated with amino acid starvation medium (Earle’s balanced salt solution EBSS medium). Trypsin–EDTA-harvested cells were spun down for 5 min at 500×*g*, 4 °C and resuspended in 400 μL 10% sucrose. Cells were then subjected to a single blast with an electric pulse of 2000 V, and the resulting cell disruptate was immediately transferred to an equal volume of ice-cold «after blast» buffer (100 mM Sodium Phosphate buffer, 2 mM EDTA, 2 mM DTT, 1.75% sucrose, pH 7.5). To measure total cellular LDH level, 150 μL of the cell disruptate was frozen at − 80 °C for at least one hour. To measure sedimentable LDH, 550 μL of the cell disruptate was mixed with 1 mL resuspension buffer (50 mM Sodium Phosphate buffer, 1 mM EDTA, 1 mM DTT, 5.9% sucrose, pH 7.5), supplemented with 0.5% BSA and 0.01% Tween 20, and spun down for 45 min at 21,000×*g*, 4 °C before frozen at − 80 °C for at least one hour. Both samples (sedimentable and total) were thawed, diluted with resuspension buffer supplemented with Triton X-405 to a final concentration of 1%, and incubated overnight on a rotator at 4 °C before spun down for 10 min at 21,000×*g*, 4 °C. LDH activity was quantified as the decline in NADH absorbance at 340 nm in an enzymatic reaction with 0.6 mM pyruvate and 0.36 mM NADH in 65 mM imidazole (pH 7.5) at 37 °C, using a multianalyzer (MaxMat PL-II, Erba Diagnostics, Mannheim, Germany). LDH sequestration activity was calculated as the percentage of sedimented LDH in treated cells subtracted the background of sedimented LDH in untreated cells and divided by the incubation time with Baf.

### Lipid peroxidation assay

Cells were grown for 24 h before 24–72 h treatments took place. Lipid peroxidation was evaluated by using Lipid peroxidation cell-based assay kit (ab243377, Abcam) according to the manufacturer`s instructions. The lipid peroxidation sensor supplemented samples were analyzed on a LSR II flow cytometer controlled by BD FACS Diva™ software. Due to the fact Melmet 1 cells are GFP-tagged only red signal was measured and compensation was performed to ensure no green signal spillover into red channel. The data was analyzed by using FlowJo software.

### In vivo studies

Melmet 1 cells (2 × 10^6^ cells/animal) were injected subcutaneously in female athymic nude *foxn1*^*nu*^ mice (6–8 weeks) bred at the Department of Comparative Medicine, Oslo University Hospital. After approximately one month (tumor volume around 100 mm^3^), the animals were randomized based on tumor size before initiation of the treatment by oral gavage with BRAFi vemurafenib, (12.5 mg/kg), AXLi BGB324, (50 mg/kg), the combination or vehicle (DMSO/methylcellulose) twice/day for 14 days. The mice were weighed and tumors measured three times a week. The tumor volume was calculated according to the formula 0.5 × length × width^2^. For molecular analysis, two animals from each treatment group were sacrificed by cervical dislocation at day 1 and day 10, 2 h after the last treatment. Tumors were than harvested and snap frozen in liquid N_2_. In the growth retardation experiments, mice were sacrificed by cervical dislocation when they were moribund or had > 20% weight loss. No mice were excluded from the analyses. The experiment was not blinded to the researcher.

The study was approved by the ethics committee, the Norwegian Food Safety Authority, (application number 7608) and conducted according to their ethical guidelines, regulations of the Federation of European Laboratory Animal Science Association (FELASA) and the ARRIVE guidelines^64^. The mice were kept under pathogen-free conditions, at constant temperature (22 ± 1 °C) and humidity (62 ± 5%), with 15 air changes/hour and a 12 h light/dark cycle. Food and water were supplied ad libitum. The mice were given paper and cardboard houses for environmental stimulation. MAK III cages were used to house the mice, and maximum 10 mice were kept in each cage. The treatment groups were mixed in the cages.

### Immunohistochemistry (IHC)

The Dako PT-link and EnVision^TM^Flex + System (Dako Glostrup, Demark, #K8012) was used for immunostaining. Formalin-fixed, paraffin-embedded tissue specimens were deparaffinised, rehydrated and target retrieval performed in one operation in a Dako PT-link and EnVision™ Flex target retrieval solution with high pH. The sections were treated with Dako EnVision Peroxidase Block for 5 min to block endogenous peroxidase. The slides were incubated with a primary antibody against AXL (R&D systems, #AF154/H1312) over night at 4 °C, then incubated with Dako EnVision™ FLEX + mouse linker for 15 min followed by incubation with Dako Envision™ FLEX/HRP for additional 30 min. The slides were further developed with DAB Chromogen (Dako, Santa Clara, CA) and counterstained with hematoxylin, dehydrated and mounted. Three semi quantitative classes were used to describe tumor cell positivity of AXL staining. The % of positively stained cells in membrane, cytoplasm or nucleus was recorded as < 10%, 10–50% and > 50%. AXL expression was evaluated by a pathologist blinded to patient clinical characteristics.

### Ex vivo drug efficacy assay

Ex vivo cultures were prepared as described previously^38^. Briefly, patient tumor tissues were mechanically disaggregated and treated for 1 h with 125 U/mL collagenase and 2.5 mg/mL DNAse (both from Sigma-Aldrich). To remove aggregates and debris the cell suspensions were filtered through 100 μM nylon Cell Strainer (BD Falcon, Franklin Lakes, NJ) and washed with ice-cold PBS. Red blood cells were removed using ACK lysing buffer (Lonza, Basel, Switzerland). Live cells were seeded out at density of 15–20 × 10^5^ cells/well in Nunc™ 96-Well Polystyrene Round Bottom Microwell plates (Thermo Fisher Scientific, Waltham, MA) in RPMI++ medium supplemented with 100 units/mL penicillin and 0.1 mg/mL streptomycin (both from Lonza) and allowed to form 3D spheroids. Drugs were immediately added to the cells and left for five days before viability was assessed using the CTG Luminescent Cell Viability Assay (Promega) and analyzed by Fluoroscan Ascent Fl (Thermo Fisher Scientific). The *ex-vivo* assay was performed once for each patient sample, with at least three technical replicates per condition.

### Statistical analysis

Two-tailed Student’s unpaired t-test was used to determine statistical significance in all in vitro analyses of cell viability, apoptosis, cell cycle distribution, protein expressions as well as in the ex vivo drug efficacy assay.

Association between AXL expression and the clinic-pathologic characteristics were analyzed by crosstabs/chi-square test.

In the in vivo experiment, statistical significance was determined by comparing area under the curve values from day 0–13 by unpaired t-test.

The differences were considered significant if *p* < 0.05, where *p ≤ 0.05; **p ≤ 0.01; and ***p ≤ 0.001.

The detection of significantly changed protein levels, identified by RPPA, between the xenografts, was based on calculations using R version 3.6.0. (The R foundation of statistical analyses). P values of less than 0.05 were considered significant (unadjusted for multiple testing).

## Supplementary Information

Supplementary Information.

## Data Availability

The part of dataset generated by RPPA analysis is included in this article (Supplementary Fig. S5). The full RPPA analysis generated datasets are available from the corresponding author on reasonable request. Identified functional enrichments within KEGG^65^ and Reactome signaling pathways were created using STRING data resource (www.string-db.org).
