# Supplementary material for: AXL inhibition improves BRAF-targeted treatment in melanoma
Source: Sci Rep. 2022 Mar 24;12:5076. doi: 10.1038/s41598-022-09078-z (PMC8948193; doi:10.1038/s41598-022-09078-z)
Supplement: Supplementary file 1 — Supplementary Information. [file 41598_2022_9078_MOESM1_ESM.pdf]

“AXL Inhibition Improves BRAF-Targeted Treatment in Melanoma”

Marta Nyakas[a,b,c], Karianne Giller Fleten[a], Mads Haugland Haugen[a], Nikolai Engedal[a], Christina Sveen[a], Inger Nina Farstad[c,d], Vivi Ann Flørenes[e], Lina Prasmickaite[a]*, Gunhild Mari Mælandsmo[a,f]* and Kotryna Seip[a]*

* The authors contributed equally.

[a] Department of Tumor Biology, Oslo University Hospital, the Norwegian Radium Hospital, Oslo, Norway;
[b] Department of Oncology, Oslo University Hospital, the Norwegian Radium Hospital, Oslo, Norway;
[c] Institute of Clinical Medicine, University of Oslo, Oslo, Norway;
[d] Department of Pathology, Division of Laboratory Medicine, Oslo University Hospital, Oslo, Norway;
[e] Department of Pathology, Oslo University Hospital, the Norwegian Radium Hospital, Oslo, Norway;
[f] Department of Medical Biology, Faculty of Health Sciences, UiT/the Arctic University of Norway, Tromsø, Norway.

Supplementary Figure S1

Bliss independence in terms of Mortality

24 hours, % of control

	AXLi, µM	Melmet 1	A375
BRAFi 0.1µM	1	0.652	0.680
	2	1.259	0.903
	3	1.004	0.972
BRAFi 1µM	1	1.145	0.747
	2	0.860	0.935
	3	1.006	0.949
BRAFi 2µM	1	0.930	0.722
	2	0.914	0.868
	3	0.965	0.915
BRAFi 3µM	1	0.828	0.583
	2	1.146	0.804
	3	0.965	0.886

72 hours, % of control

	AXLi, µM	Melmet 1	A375
BRAFi 0.1µM	1	0.856	1.084
	2	0.848	1.070
	3	0.996	0.998
BRAFi 1µM	1	0.900	1.009
	2	0.828	1.005
	3	1.006	1.009
BRAFi 2µM	1	0.849	1.011
	2	0.887	1.004
	3	1.008	1.011
BRAFi 3µM	1	0.876	1.000
	2	0.812	0.998
	3	1.014	1.009

Additivity
Synergy
Antogonism

Synergy < 0.9 - Additivity - 1.1 < Antagonism

Supplementary Figure S1. Bliss independence scores in differently treated melanoma cell lines. Melmet 1 and A375 cells were treated with different doses of BRAFi, AXLi or a combination of both as indicated for 24 or 72 hours. Bliss independence in terms of Mortality was calculated as follow: $=((M_A+M_B)-(M_A*M_B))/M_{AB}$, where A and B is cell mortality related to control. < 0.9 indicates Synergy, 0.9 – 1.1 indicates Additivity and > 1.1 indicates Antagonism. Red squares highlight the chosen BRAFi and AXLi doses for mono- and combination treatments presented as cell viability data in Fig. 3c.

Supplementary Figure S2_Part 1 out of 4

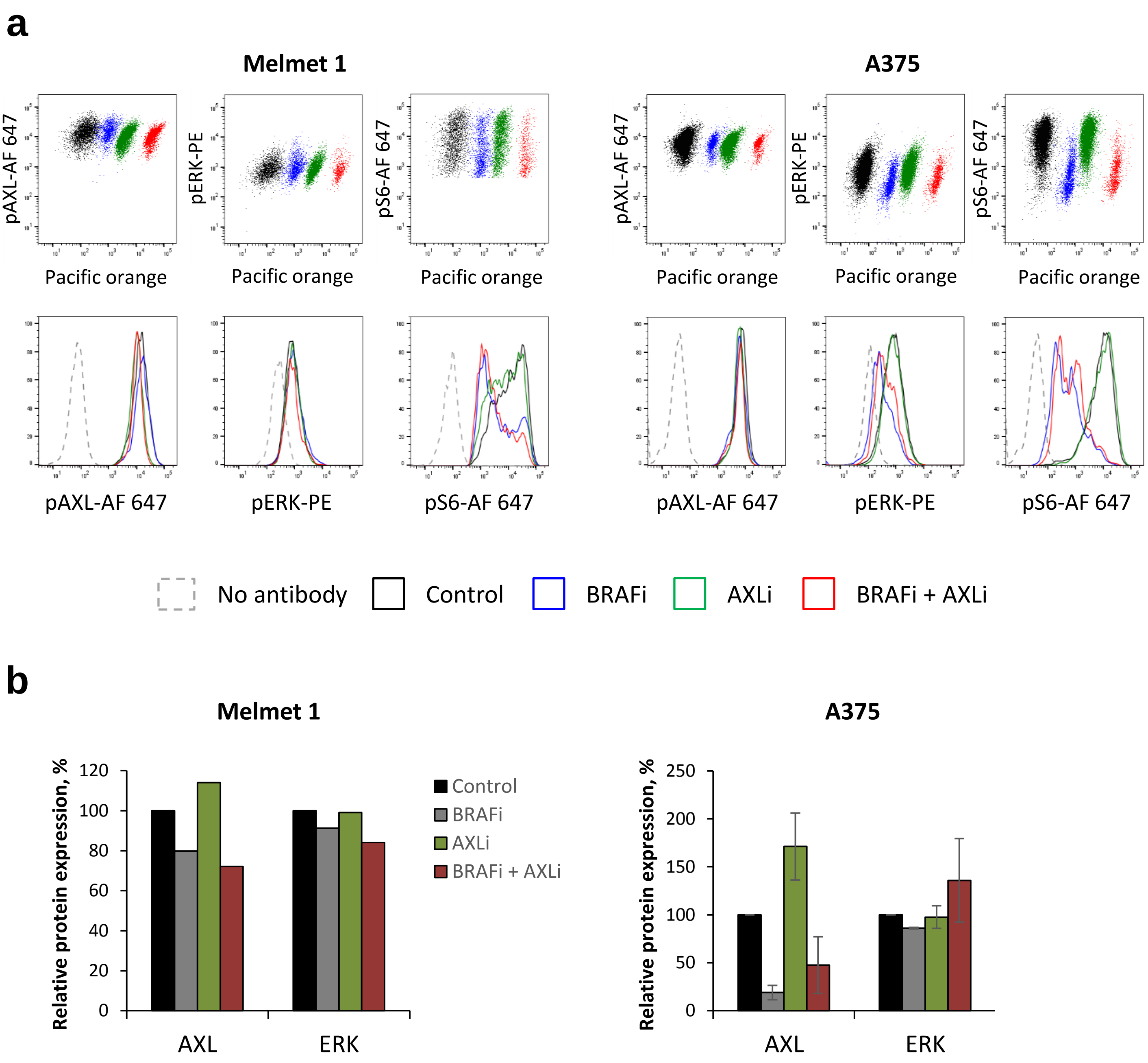

Supplementary Figure S2. AXL, ERK and S6 protein activity and expression in melanoma cells. Melanoma cells were treated with 2 μM BRAFi, 2 μM AXLi or a combination of both for 48 hours and analyzed by FLOW (**a**) or SWI (**b-e**). **(a)** Representative dot plots and histograms of barcoded samples indicating protein activity. AF – Alexa Fluor. **(b)** The levels of the total proteins (after normalization to the loading control, β-actin) presented as relative to respective non-treated controls set to 100%. n=1 for Melmet 1 and Average ± St. Dev, n=2 for A375. **c-e**) peaks of indicated proteins from SWI in one analyzed set of Melmet 1 (**c**) and two sets of A375 (**d** and **e**). The protein levels were calculated by integration of the area below peaks (colored area) detected by chemilumninescence.

Supplementary Figure S2_Part 2 out of 4

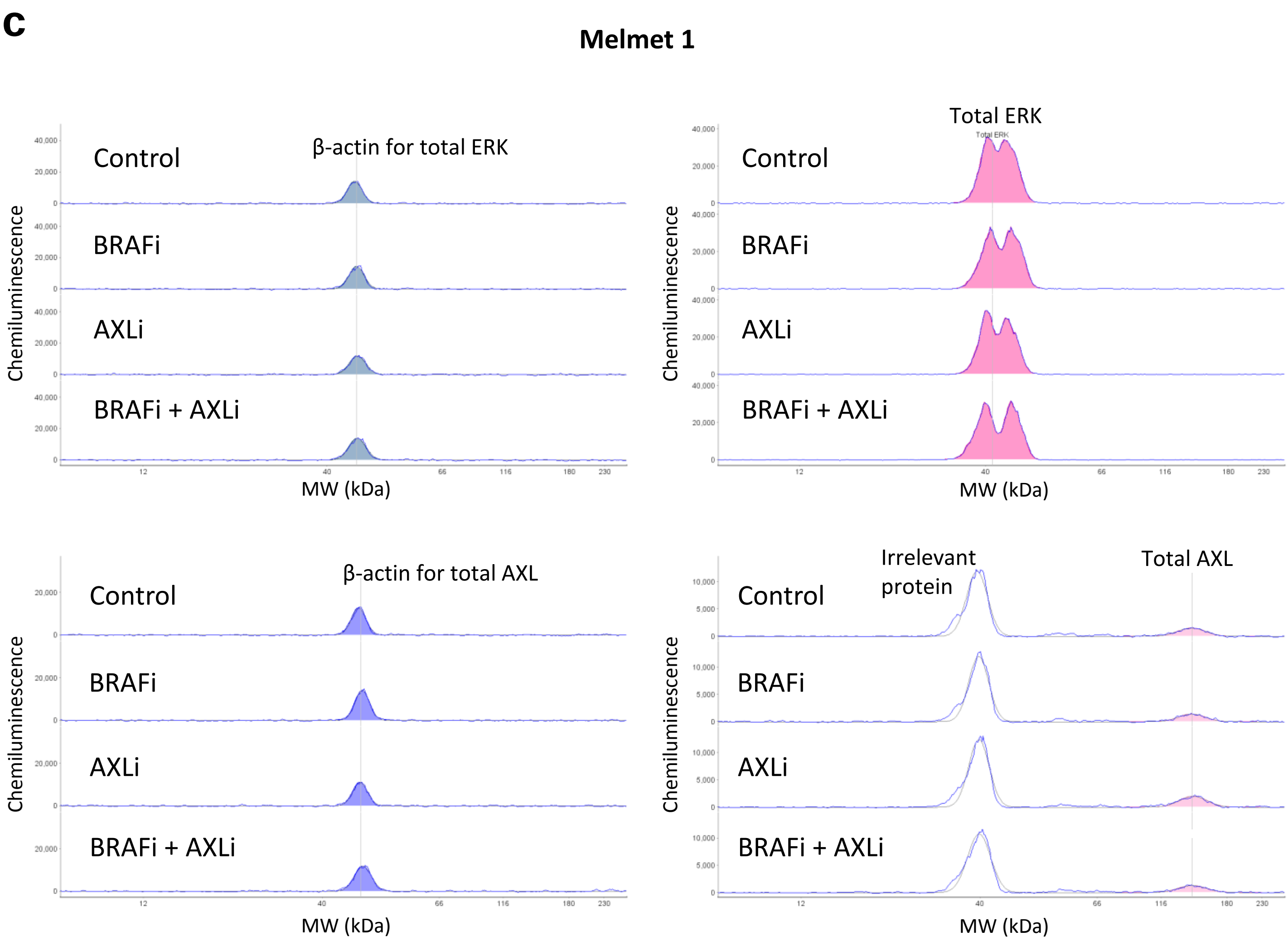

Supplementary Figure S2_Part 3 out of 4

d

A375 set No. 1

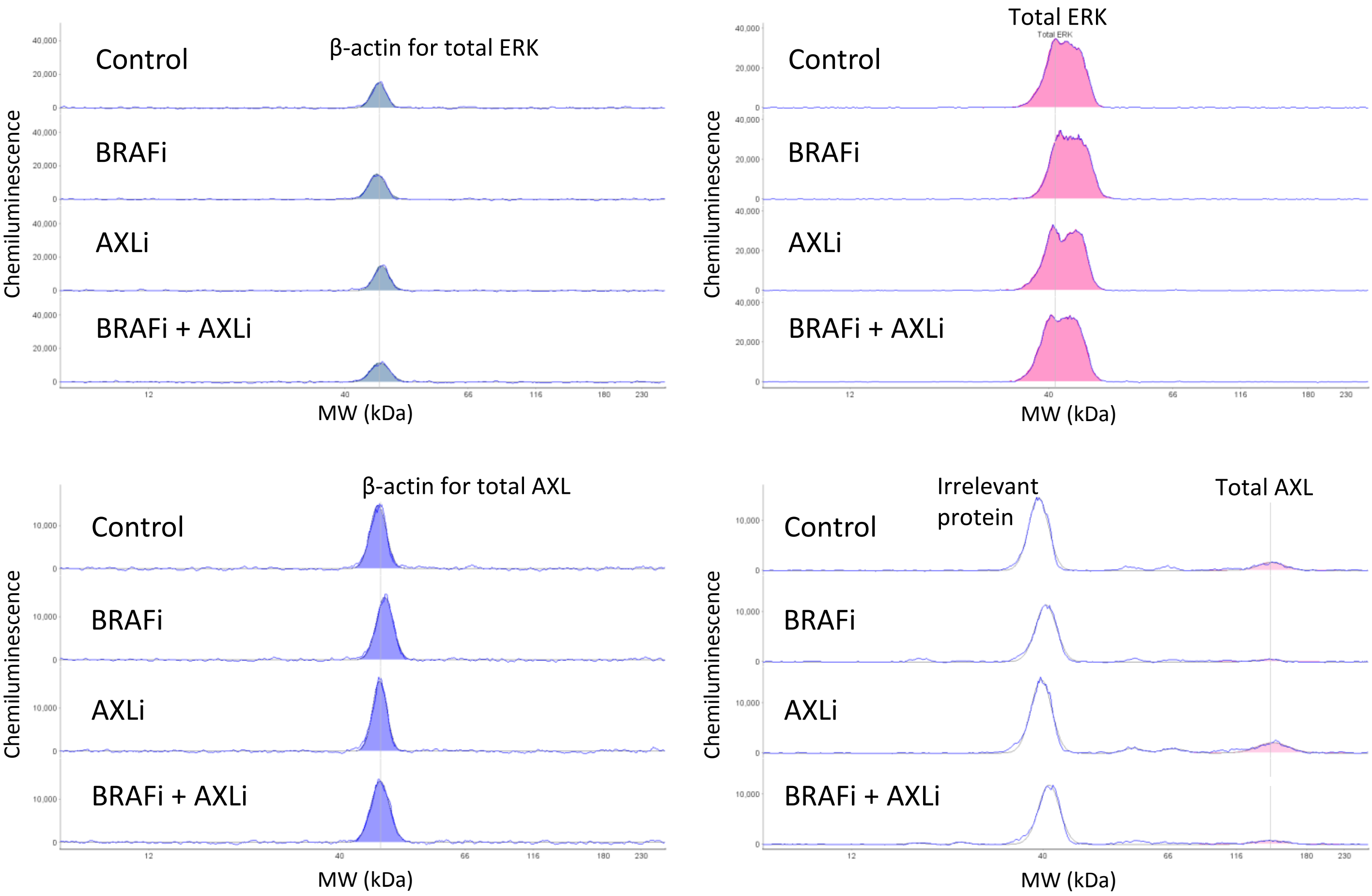

Supplementary Figure S2_Part 4 out of 4

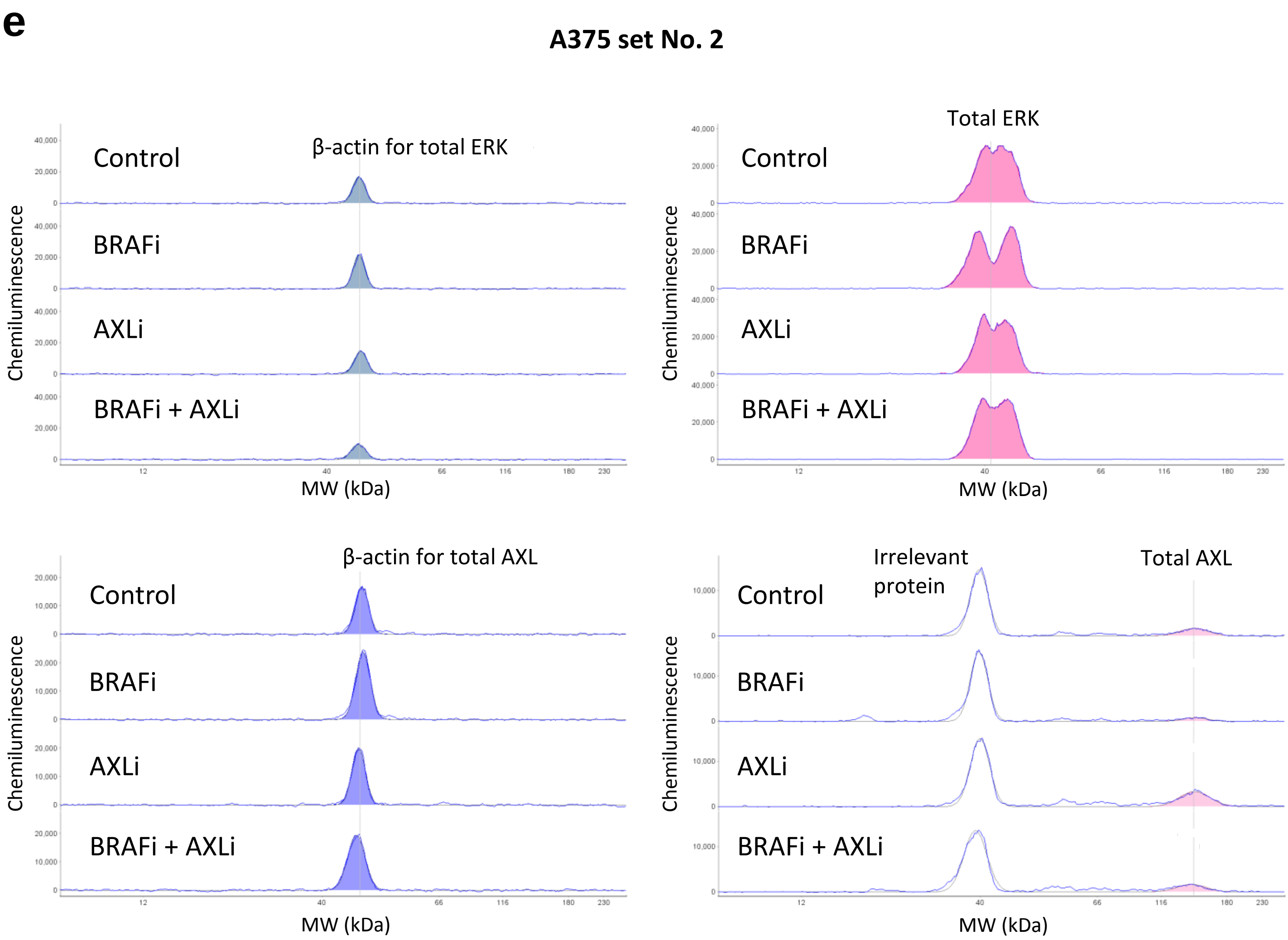

Supplementary Figure S3

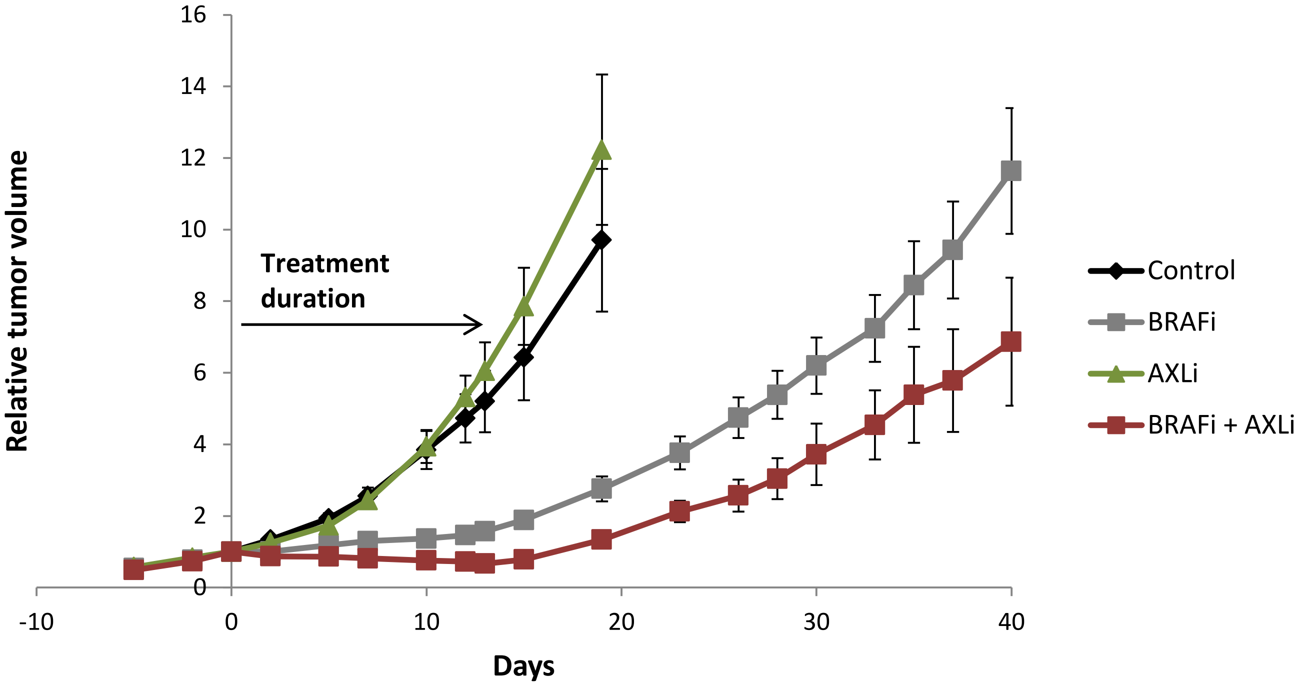

Supplementary Figure S3. Tumor cells re-grow after treatment withdrawal. Subcutanous Melmet 1 tumors were treated with BRAFi: 12.5 mg/kg, AXLi: 50 mg/kg, the combination or vehicle (DMSO/methylcellulose) twice/day for 14 days. The relative tumor volume over time is presented.

Supplementary Figure S4_Part 1 out of 5

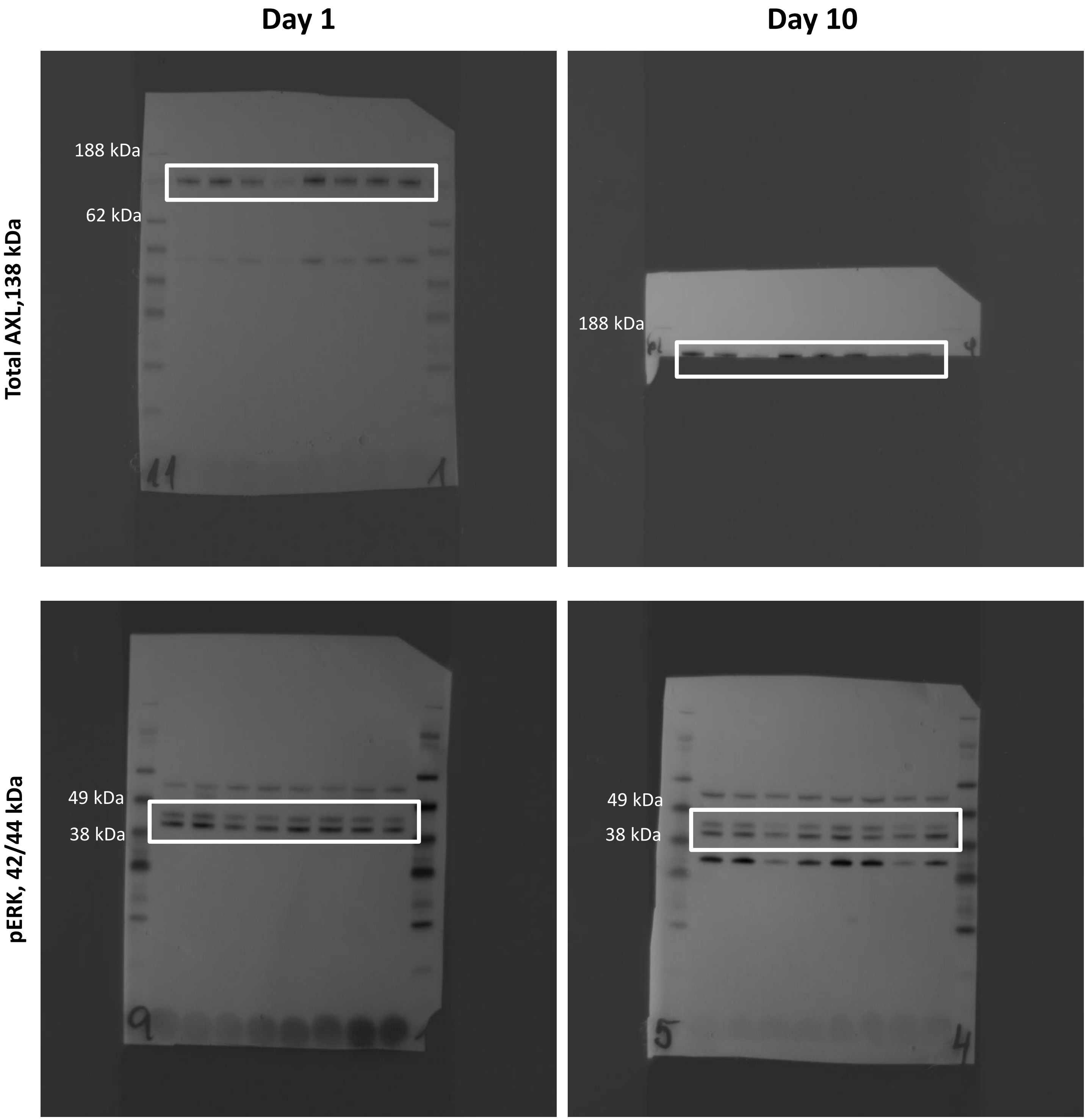

Supplementary Figure S4. Uncropped Western immunoblots of indicated proteins used in Figure 6b, c. Merged chemiluminesence and colorimetric image to show the position of the molecular weight markers. The order of samples is the same as in the Figure 6b, c. Protein bands used in the main figure are shown within rectangle.

Supplementary Figure S4_ Part 2 out of 5

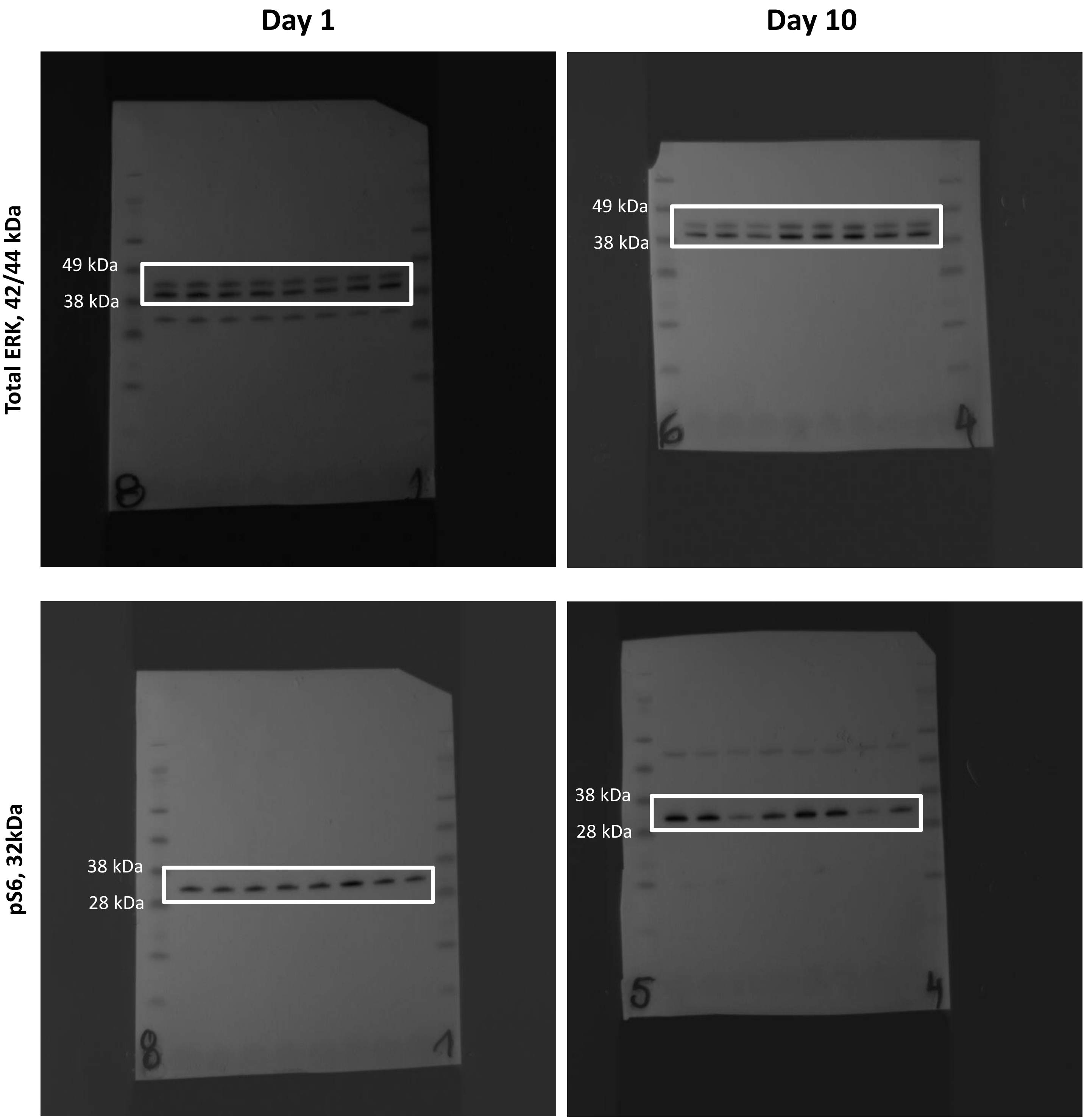

Supplementary Figure S4_ Part 3 out of 5

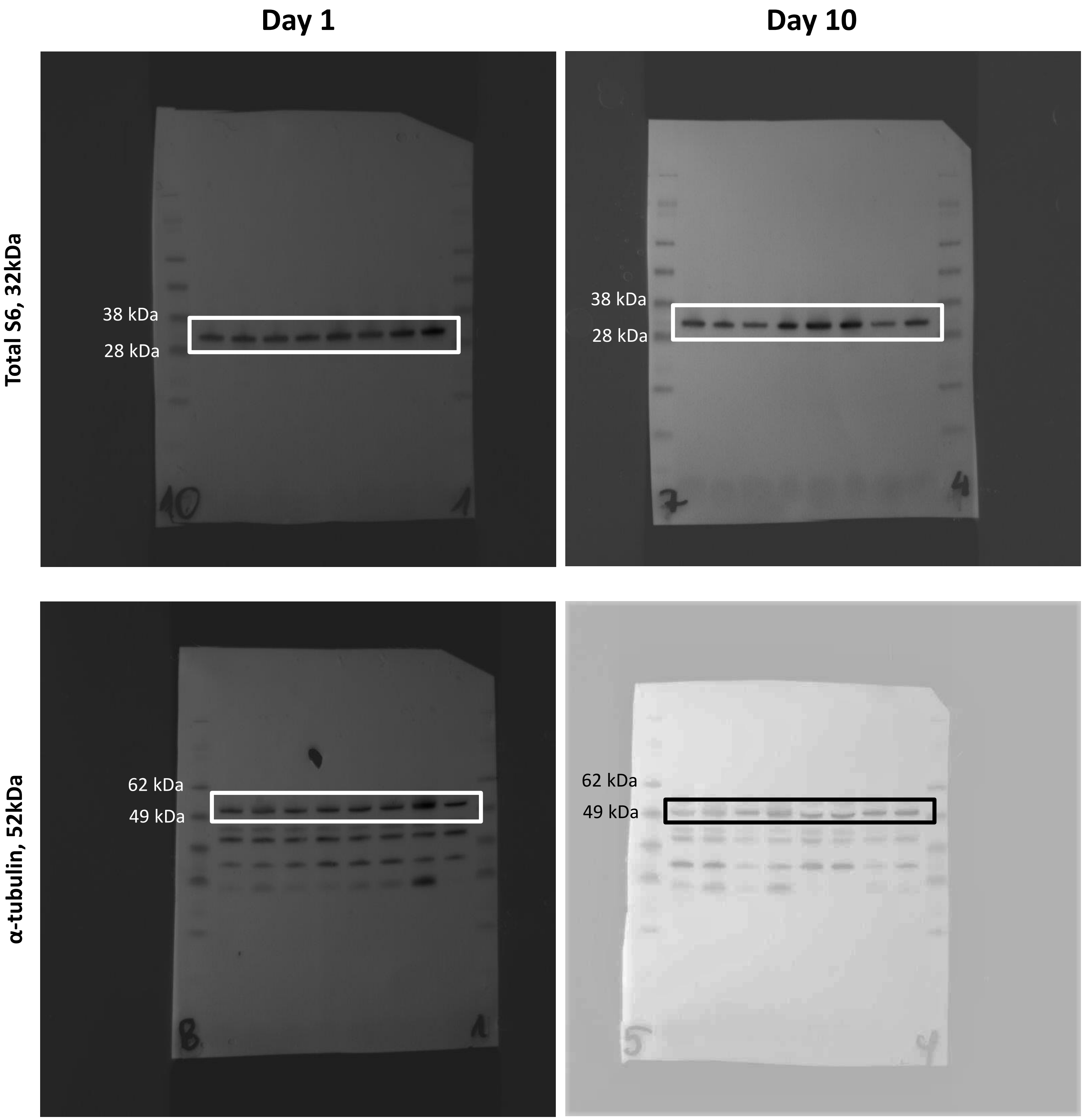

Supplementary Figure S4_Part 4 out of 5

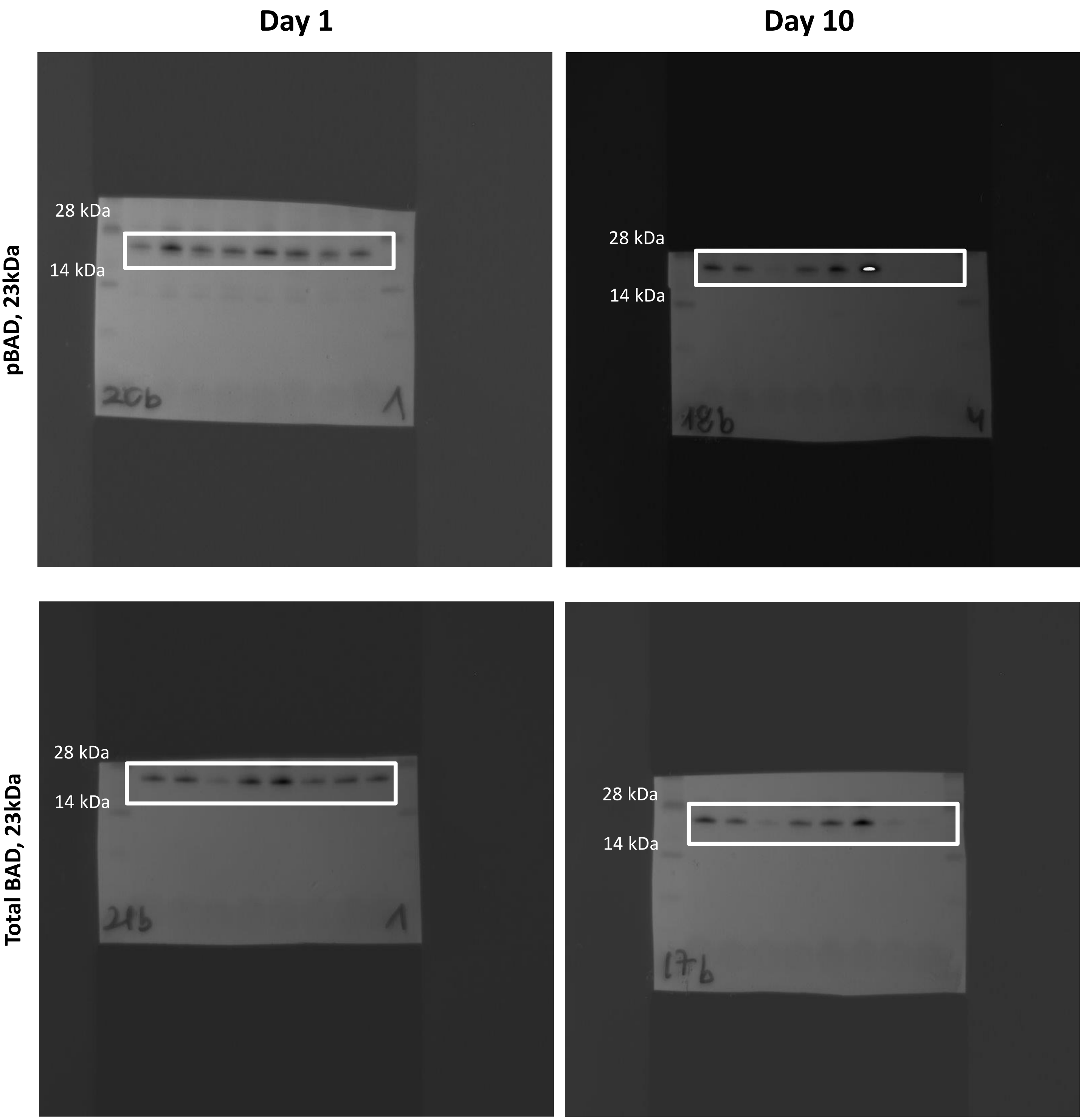

Supplementary Figure S4_Part 5 out of 5

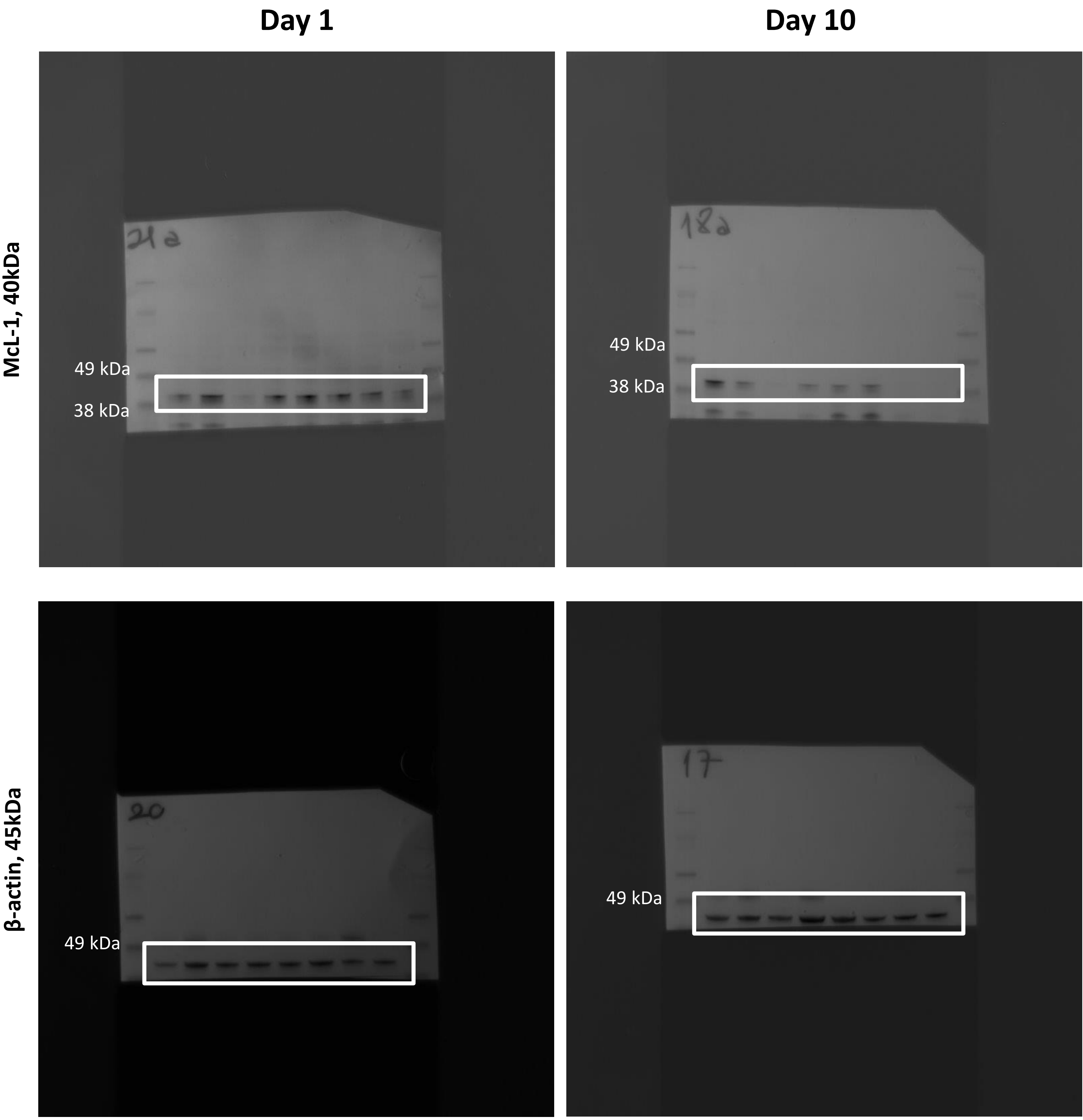

Supplementary Figure S5

AXLi *vs* Control (**AXLi effect**)

Protein	Log2 FC	p value
LC3A-B	0.525	0.007
B7-H4	0.356	0.000
Rab25	0.287	0.025
GCLM	0.255	0.025
Raptor	0.216	0.041
p27-Kip-1	0.132	0.040
ACC_pS79	-0.154	0.037
JNK2	-0.389	0.045
Stat3_pY705	-0.464	0.026
PMS2	-0.498	0.012
mTOR	-0.556	0.005
Ets-1	-0.618	0.032
Akt_pT308	-0.692	0.003
TFRC	-0.699	0.045
PKC-b-II_pS660	-0.776	0.031
b-Catenin	-1.222	0.010
Akt_pS473	-1.263	0.006
eEF2	-1.529	0.034

Combination *vs* BRAFi (**Additive AXLi effect**)

Protein	Log2 FC	p value
SHP-2_pY542	0.511	0.028
LC3A-B	0.432	0.008
B7-H4	0.325	0.001
N-Cadherin	-0.177	0.033
ACC_pS79	-0.250	0.017
Caveolin-1	-0.516	0.050
mTOR_pS2448	-0.730	0.040
Cyclin-B1	-0.758	0.047
SLC1A5	-0.895	0.013

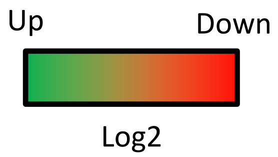

Supplementary Figure S5. Significantly modulated proteins upon AXLi mono- treatment and as additive effect in combination with BRAFi. Melmet 1 tumors treated with BRAFi: 12.5 mg/kg, AXLi 50 mg/kg, the combination or vehicle (DMSO/methylcellulose) for ten days (twice a day) were subjected to RPPA analysis and significant AXLi-induced protein changes either upon mono-treatment "AXLi *vs* control" (left) or as an additive effect when combined with BRAFi "Combination *vs* BRAFi" (right) is presented. Proteins ranked on Fold Change (FC). Green and red colors indicate up-regulated and down-regulated proteins, respectively.

Supplementary Figure S6_Part 1 out of 2

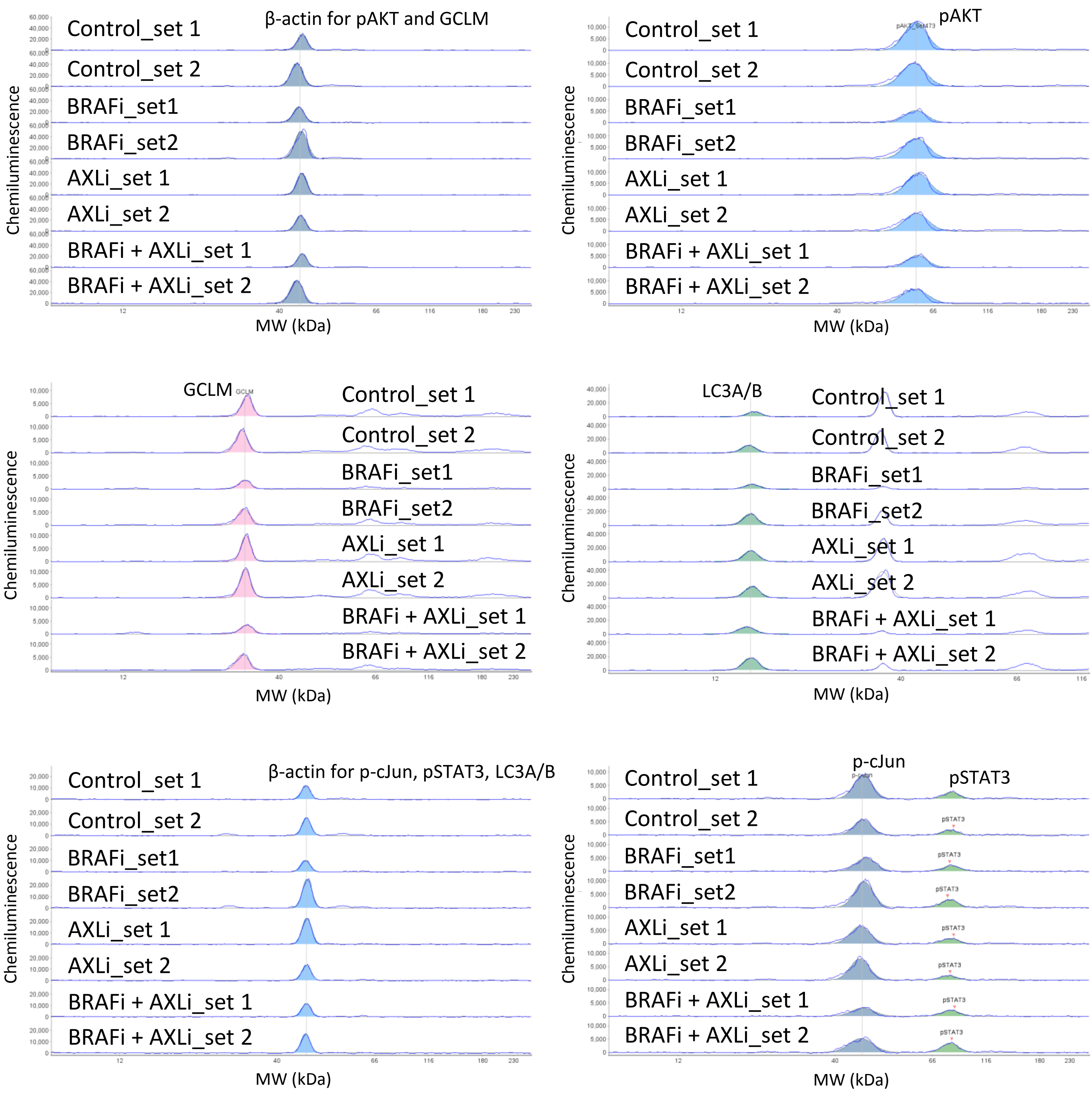

Supplementary Figure S6. The peaks of indicated proteins detected by SWI that were used to calculate the protein expression levels presented in Figure 7c. Melmet 1 tumors were treated with BRAFi: 12.5 mg/kg, AXLi 50 mg/kg, the combination or vehicle (DMSO/methylcellulose) for ten days (twice a day). The protein levels were calculated by integration of the area below peaks (colored area) detected by chemilumninescence.

Supplementary Figure S6_Part 2 out of 2

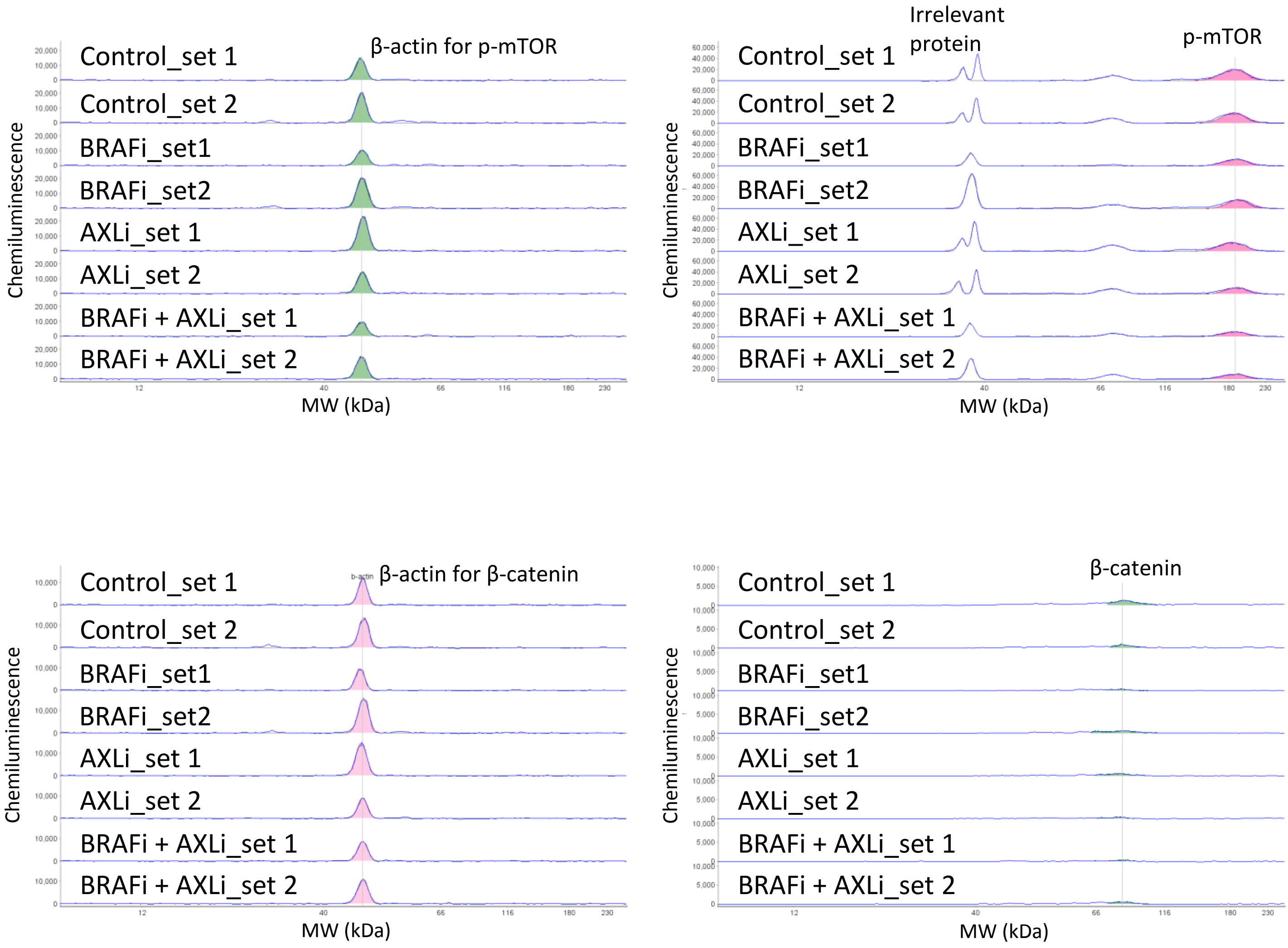

Supplementary Figure S7

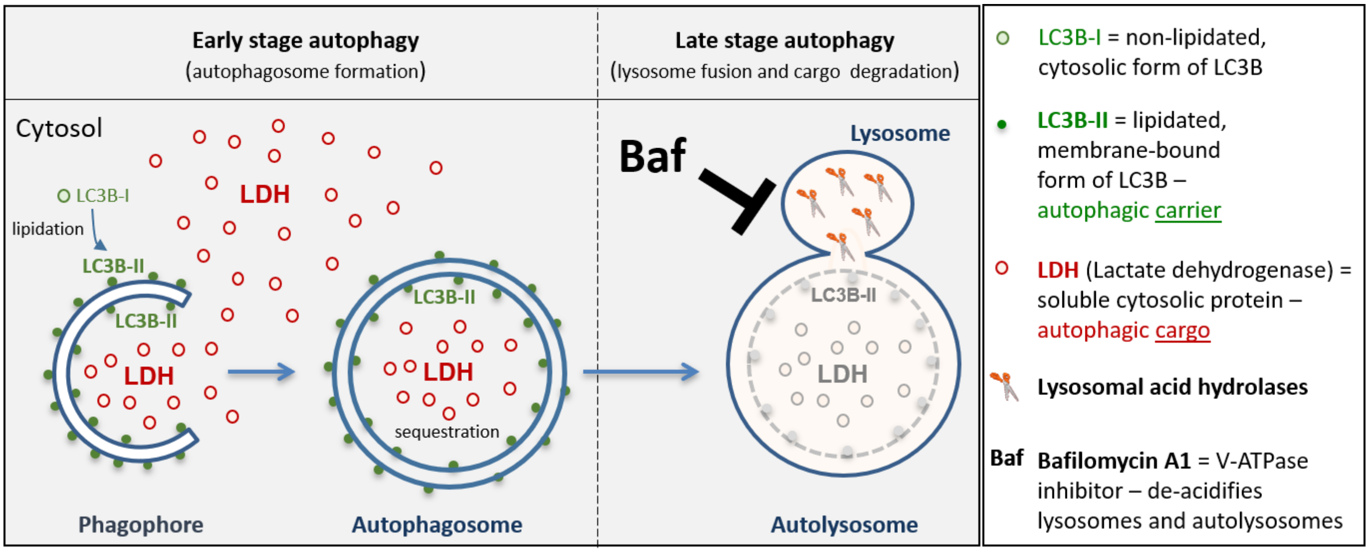

Supplementary Figure S7. Illustration of the principles for the "LC3B flux assay" (used to measure autophagic membrane flux) and the "LDH sequestration assay" (used to measure autophagic cargo flux) employed in the main Figure 8. At the early stages of autophagy (left), the cytosolic protein LC3B-I is conjugated to lipids on both sides of the double-membrane autophagosome precursor structure (the «phagophore»), thus generating the membrane-bound form LC3B-II. LC3B-II on the inner membrane stays attached all the way through the pathway to the autophagosome-lysosome fusion step, where after it is degraded by lysosomal proteases (right). The autophagic degradation of LC3B-II can be blocked by treatment with the V-ATPase inhibitor Bafilomycin A1 (Baf), which de-acidifies lysosomes and thereby inactivates the lysosomal acid hydrolases. Changes in LC3B-II levels can therefore be used as a proxy measure of autophagic ***membrane flux*** through the pathway. If a treatment increases LC3B-II levels in the absence of Baf but not in its presence, it most likely acts to reduce autophagic flux at a late stage in the autophagy pathway. If a treatment is able to increase LC3B-II in the presence of Baf, but not in its absence, it is interpreted to activate autophagy at the early stages of the pathway [1]. Whereas the LC3B flux assay can indicate LC3B-associated autophagic membrane flux, it does not provide any direct information about the actual autophagic ***cargo flux***. The lactate dehydrogenase (LDH) sequestration assay can be used to quantify the degree of intracellular sequestration of the soluble cytosolic enzyme LDH into autophagosomes. When the phagophore expands and closes in on itself to form the autophagosome (left), part of the cytosol is included, and thus LDH is sequestered along with other autophagic cargo into the autophagosome. With the same principle as for the LC3B flux assay, the influence of a particular treatment on the flux of LDH through the autophagic pathway can be measured by comparing its sequestration into autophagosomes (and thus its accumulation into the sedimentable cellular fraction) in the absence and presence of Baf. If a treatment increases the levels of sequestered LDH in the absence of Baf but not in its presence, it most likely acts to reduce autophagic flux at a step after autophagosome formation, i.e. at a late stage in the autophagy pathway. If a treatment is able to increase the levels of sequestered LDH in the presence of Baf but not in its absence, it is interpreted to activate autophagy at an early stage of the pathway, resulting in increased autophagosome formation [1, 2].

1. Klionsky et al., Guidelines for the use and interpretation of assays for monitoring autophagy (4th edition). Autophagy. 2021;17(1):1-382; 2. Luhr et al., The Lactate Dehydrogenase Sequestration Assay - A Simple and Reliable Method to Determine Bulk Autophagic Sequestration Activity in Mammalian Cells. Journal of visualized experiments: JoVE. 2018(137).).

Supplementary Figure S8

Sample set No. 1_used for both the representative image and quantification

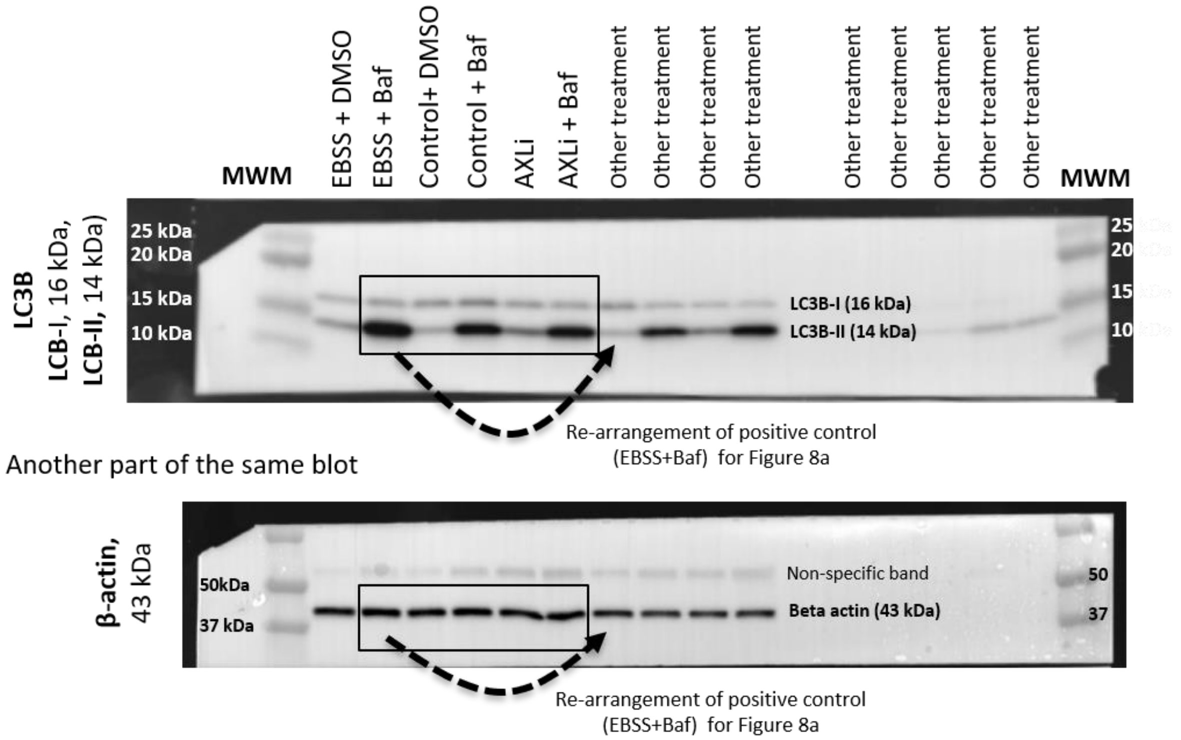

Sample set No. 2_used for quantification only

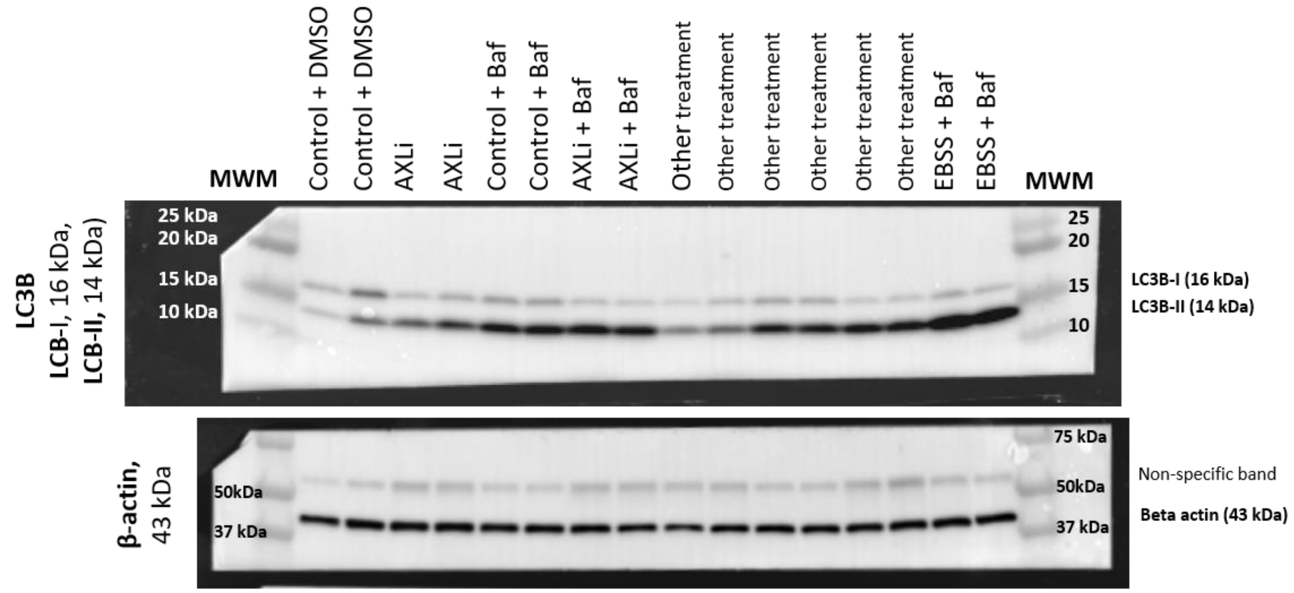

Supplementary Figure S8. Uncropped Western immunoblots of indicated proteins used in Figure 8a. Merged chemiluminesence and colorimetric image to show the position of the molecular weight markers. Protein bands used in the main figure are shown within rectangle.

Supplementary Figure S9

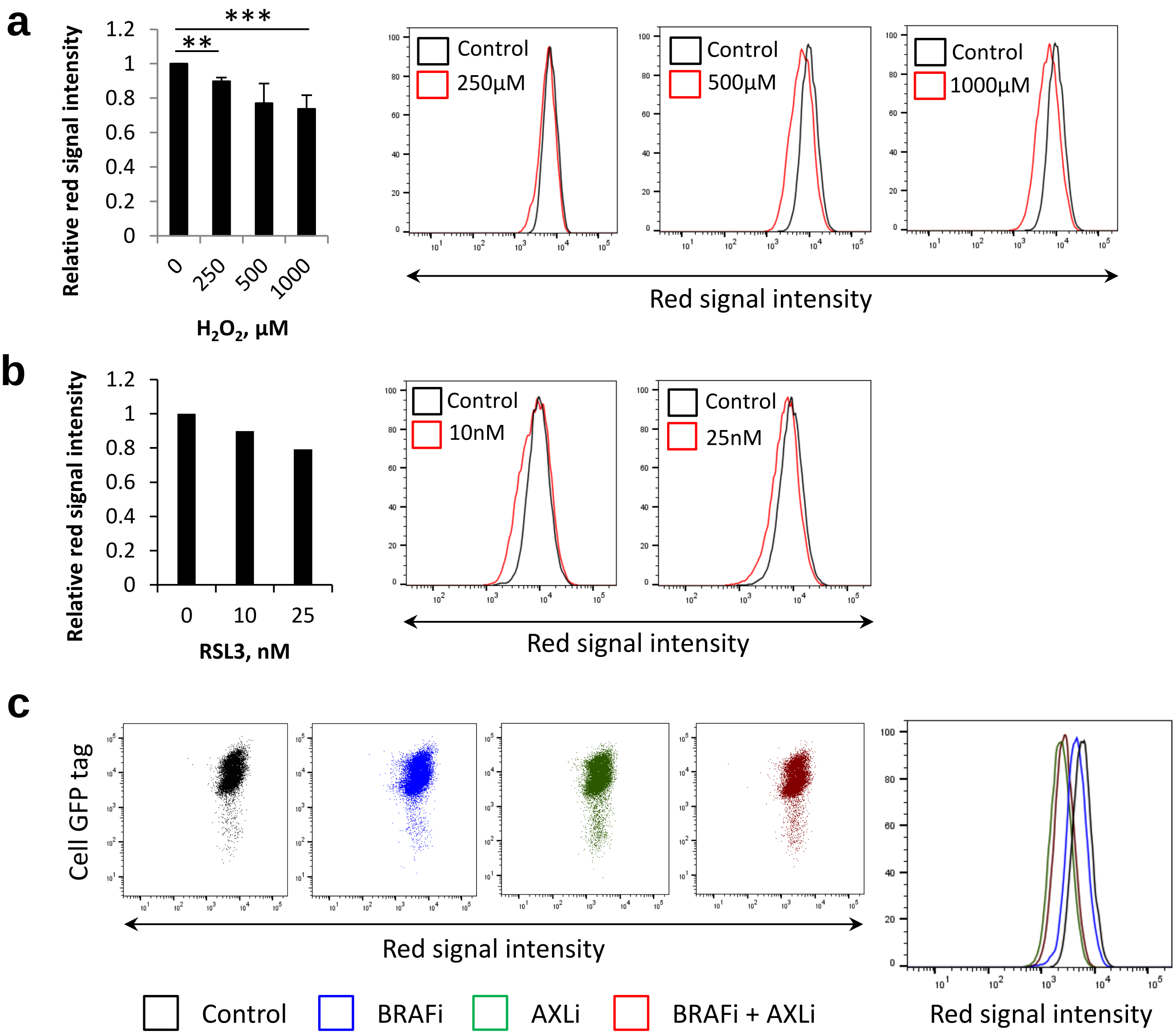

Supplementary Figure S9. Detection of lipid peroxidation in mono- and combination treatment with BRAFi and AXLi. Red signal intensities of lipid peroxidation sensor measured by FLOW (recording $\geq 1 \times 10^4$ cells) in differently treated Melmet 1 cells. **a, b**) Melmet 1 cells were treated with increasing concentration of hydrogen peroxide (H_2O_2) and ferroptosis inducer RSL3 for 0.5 and 24 hours, respectively. Relative signal mean intensities were calculated and related to respective controls. Representative histograms of indicated samples are shown. Average ± SEM, n=3 for H_2O_2 and n=1 for RSL3 data. **, $p \leq 0.01$; and ***, $p \leq 0.001$. **c**) Melmet 1 cells were treated with 2 µM BRAFi, 1 µM AXLi or a combination of both, DMSO for control for 72 hours. Representative dot plots and histograms of differently treated samples are shown. Red signal intensities were measured in PE channel.

Supplementary Figure S10

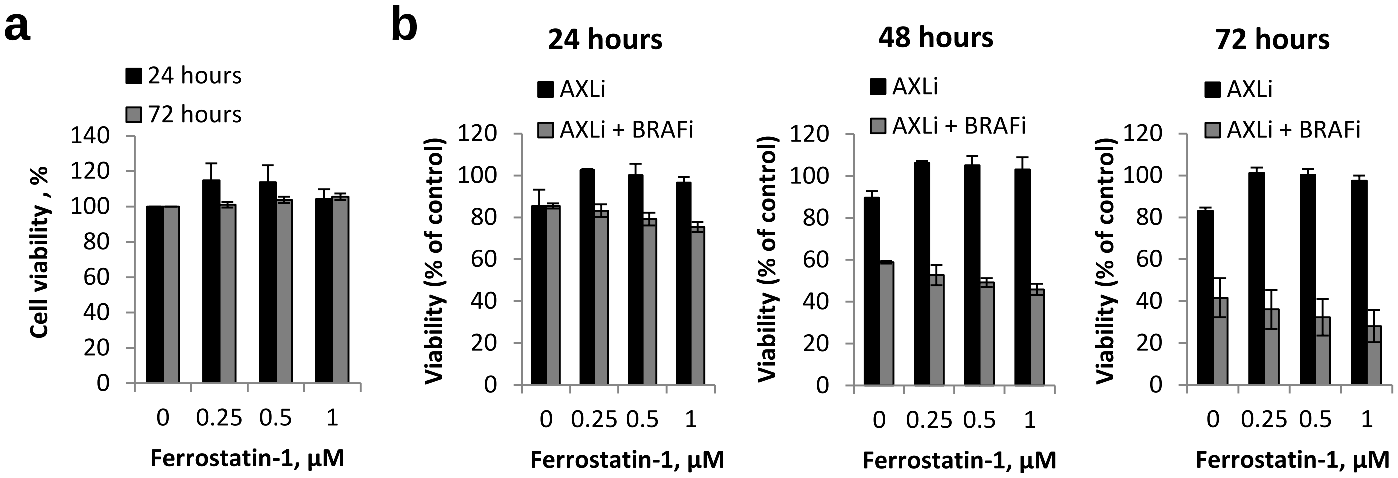

Supplementary Figure S10. Effect of ferroptosis inhibitor on cell viability in mono- and combination treatment with BRAFi and AXLi. Cell viability of Melmet 1 cells treated with different concentrations of the ferroptosis inhibitor ferrostatin - 1 (Fer-1), as indicated in mono- (**a**) or in combination treatment with either 0.5 µM AXLi alone or 2 µM BRAFi and 0.5 µM AXLi for 24, 48 or 72 hours **(b)**. The treatment effect was scored by CellTiterGlo assay and presented as % relative to the respective non-treated controls set to 100%. (**a**) Average ± SEM, n=3. (**b**) Average ± St. Dev. for 24 and 48 hours, n=1 and Average ± SEM for 72 hours, n=3.

Supplementary Table S1. AXL expression and characteristics of the patients. *Data are shown as number of patients and percentage of known data.

		AXL Expression		
**Characteristics**	**Variable**	**Low < 10% n (%)***	**High ≥ 10% n (%)***	**P value**
Age, n = 72	< 60 years	18 (25)	17 (23.6)	.650
	≥ 60 years	21 (29.2)	16 (22.2)	
Gender, n = 72	Female	11 (15.3)	12(16.7)	.459
	Male	28 (38.9)	21 (29.2)	
Melanoma type, n = 57	Superficial	16 (28.1)	12 (21.1)	.705
	Nodular	18 (31.6)	11 (19.3)	
Tumor thickness, n = 60	T1 ≤ 1.00 mm	2 (3.3)	3 (5)	.316
	T2 1.01 - 2.00 mm	10 (16.7)	11 (18.3)	
	T3 2.01 - 4.00 mm	8 (13.3)	4 (6.7)	
	T4 > 4 mm	13 (21.7)	9 (15)	
Ulceration, n = 52	No	16 (30.8)	11 (21.2)	.812
	Yes	14 (26.9)	11 (21.2)	
Primary location, n = 65	Head-neck	3 (4.6)	2 (3.1)	.514
	Back-shoulders	15 (23.1)	11 (16.9)	
	Front torso	8 (12.3)	6 (9.2)	
	Arms	3 (4.6)	2 (3.1)	
	Legs	7 (10.8)	8 (12.3)	
Time primary lesion to local metastasis, n = 65	Months	36 (55.5)	29 (44.6)	.286
$BRAF^{V600}$ mutation,	Negative	6 (15)	11 (27.5)	.530

n = 40				
	Positive	6 (15)	17 (42.5)	
NRAS mutation, n = 40	Negative	9 (22.5)	21 (52.5)	1.0
	Positive	3 (7.5)	7 (17.5)	

Supplementary Table S2. Top most affected signaling pathways in differently treated Melmet 1 tumors

**BRAFi *vs* Control (BRAFi effect)**		
**KEGG Pathways**	**Strength**	**FDR**
Central carbon metabolism in cancer	1.83	0.0145
Adipocytokine signaling pathway	1.8	0.0145
Chronic myeloid leukemia	1.76	0.0145
ErbB signaling pathway	1.72	0.0145
Endocrine resistance	1.66	0.0145
Prostate cancer	1.65	0.0145
HIF-1 signaling pathway	1.65	0.0145
Insulin resistance	1.61	0.0145
AMPK signaling pathway	1.56	0.0145
Proteoglycans in cancer	1.52	0.0058
Insulin signaling pathway	1.51	0.0145
Cell adhesion molecules (CAMs)	1.5	0.0145
**Reactome Pathway**	**Strength**	**FDR**
Constitutive Signaling by AKT1 E17K in Cancer	2.22	0.0067
VEGFR2 mediated vascular permeability	2.21	0.0067
PI3K/AKT Signaling in Cancer	1.88	0.0014
Costimulation by the CD28 family	1.85	0.0115
Macroautophagy	1.82	0.0115
Cell-Cell communication	1.53	0.0204

Cell surface interactions at the vascular wall	1.51	0.0215

**Combination *vs* AXLi (Additive BRAFi effect)**		
**KEGG Pathways**	**Strength**	**FDR**
Apoptosis - multiple species	1.76	0.0048
Renal cell carcinoma	1.59	0.0011
Type II diabetes mellitus	1.59	0.0078
Progesterone-mediated oocyte maturation	1.58	0.00053
HIF-1 signaling pathway	1.56	0.00053
Insulin resistance	1.52	0.00053
ErbB signaling pathway	1.51	0.0017
**Reactome Pathway**	**Strength**	**FDR**
Activation, myristolyation of BID and translocation to mitochondria	2.65	0.0043
Polo-like kinase mediated events	2.05	0.0212
Cyclin A/B1/B2 associated events during G2/M transition	1.85	0.0212
MAPK targets/ Nuclear events mediated by MAP kinases	1.76	0.0212
RET signaling	1.67	0.0216
Interleukin-3, Interleukin-5 and GM-CSF signaling	1.58	0.0269

**AXLi *vs* Control (AXLi effect)**		
**KEGG Pathways**	**Strength**	**FDR**
Ferroptosis	1.94	0.0001
Autophagy - other	1.88	0.0015
Adipocytokine signaling pathway	1.82	0.0000166
Pancreatic cancer	1.79	0.0000166
Longevity regulating pathway - multiple species	1.75	0.00027
Acute myeloid leukemia	1.72	0.0003
Prolactin signaling pathway	1.7	0.00032
Type II diabetes mellitus	1.7	0.0029
AMPK signaling pathway	1.68	0.00000694
HIF-1 signaling pathway	1.67	0.0000382
EGFR tyrosine kinase inhibitor resistance	1.65	0.00042
Insulin signaling pathway	1.63	0.00000694
Insulin resistance	1.63	0.0000447
ErbB signaling pathway	1.62	0.00046
Colorectal cancer	1.61	0.00046
Longevity regulating pathway	1.59	0.00047
Autophagy - animal	1.57	0.0000698
Endocrine resistance	1.56	0.00053
Choline metabolism in cancer	1.55	0.00055
AGE-RAGE signaling pathway in diabetic complications	1.55	0.00055

Central carbon metabolism in cancer	1.55	0.0053
Non-small cell lung cancer	1.54	0.0053
Fc epsilon RI signaling pathway	1.54	0.0053
Th17 cell differentiation	1.53	0.00056
Glioma	1.53	0.0053
Renal cell carcinoma	1.53	0.0053
Toxoplasmosis	1.5	0.00065
**Reactome Pathway**	**Strength**	**FDR**
CD28 dependent PI3K/Akt signaling	2.02	0.006
mTORC1-mediated signalling	2.02	0.006
HSF1-dependent transactivation	2	0.006
mTOR signalling	1.95	0.00066
Constitutive Signaling by AKT1 E17K in Cancer	1.95	0.006
VEGFR2 mediated vascular permeability	1.93	0.006
Energy dependent regulation of mTOR by LKB1-AMPK	1.9	0.006
Regulation of TP53 Degradation	1.82	0.006
Intrinsic Pathway for Apoptosis	1.73	0.0078
Macroautophagy	1.72	0.0015
Signaling by PTK6	1.65	0.0015
TP53 Regulates Metabolic Genes	1.62	0.0023
Regulation of PTEN gene transcription	1.59	0.0119

Combination *vs* BRAFi (Additive AXLi effect)		
**KEGG Pathways**	**Strength**	**FDR**
Ferroptosis	2.04	0.0115
p53 signaling pathway	1.81	0.0115
Longevity regulating pathway	1.69	0.0133
Thyroid hormone signaling pathway	1.58	0.0179
Natural killer cell mediated cytotoxicity	1.54	0.0179
Osteoclast differentiation	1.54	0.0179
Fluid shear stress and atherosclerosis	1.51	0.0179
Apoptosis	1.51	0.0179
**Reactome Pathway**	**Strength**	**FDR**
Intrinsic Pathway for Apoptosis	2	0.0243
Macroautophagy	1.82	0.0243
TP53 Regulates Metabolic Genes	1.72	0.0243
PI3K/AKT Signaling in Cancer	1.71	0.0243
Protein folding	1.65	0.0243
Interleukin-4 and Interleukin-13 signaling	1.61	0.0243
Cell surface interactions at the vascular wall	1.51	0.0243

Melmet 1 tumors were treated with BRAFi: 12.5 mg/kg, AXLi 50 mg/kg, the combination or vehicle (DMSO/methylcellulose) for ten days, twice a day. Significantly modulated proteins between differently treated tumors were analyzed by RPPA analysis and further subjected to STRING database (www.string-db.org). Top identified functional enrichments within KEGG (Kanehisa M. *et al.*, Nucleic Acids Res., 2021) and Reactome signaling pathway databases were ranked based on strength (set to be above 1.5). Strength and False Discovery Rate (FDR) describe how large and significant, respectively, the enrichment effect is.
